# Development of a strategy for sampling, preservation, and analysis of classical and emerging flame retardants and plasticizers in air samples

**DOI:** 10.1007/s00216-025-06057-x

**Published:** 2025-09-02

**Authors:** Judith Desmet, Maria A. Aretaki, Mar Viana, Ethel Eljarrat

**Affiliations:** 1 Institute of Environmental Assessment and Water Research, (IDAEA)-CSIC, Jordi Girona 18-26, 08034 Barcelona, Spain; 2 Pollution Prevention Unit, Ministry for Ecological Transition, Pza. San Juan de La Cruz 10, 28071 Madrid, Spain

**Keywords:** Analyte stability, Emerging contaminants, Environmental monitoring, Indoor pollution

## Abstract

This study presents a methodology for sampling, preserving, and analyzing 15 organophosphate esters (OPEs), 11 phthalate esters (PEs), and 6 alternative plasticizers (APs) in indoor air. Accurate quantification is essential, given their widespread use in household items and adverse health effects associated with exposure. Solid-phase extraction (SPE) cartridges, collecting both gaseous and particulate phases, connected to a low-volume pump were used for the collection of air samples. Stability tests confirm that storing samples at 4 °C is most optimal to maintain analyte integrity for at least 2 weeks. The analytes were extracted and then purified online using turbulent flow chromatography, before analysis via liquid chromatography-tandem mass spectrometry (TFC-LC–MS/MS). Quantification was carried out by applying the isotopic dilution method with labelled standards. Recoveries ranged from 50 to 136%, with reproducibility below 21%. The relative standard deviation (RSD) varied from 2 to 31%, only exceeding the 20% threshold for certain OPEs at lower concentrations. Limits of detection (mLODs) varied between 0.02–0.89 ng/m^3^, 0.02–20.0 ng/m^3^, and 0.07–1.47 ng/m^3^ for OPEs, PEs, and APs, respectively. The study demonstrates that this methodology is effective, cost-efficient, and suitable for monitoring a total of 33 flame retardants and plasticizers in indoor air. It minimizes material costs and sample handling time by allowing direct analysis without pretreatment. Results from indoor air samples collected in a case study revealed that OPEs attributed to less than 20% of the chemical profile, while triethyl citrate (TEC) was detected at concentrations up to 28.9 ng/m^3^, confirming the method’s applicability for the added APs.

**Graphical Abstract:**

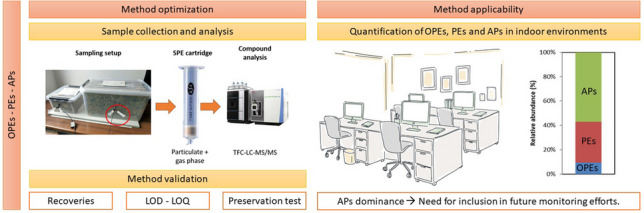

**Supplementary Information:**

The online version contains supplementary material available at 10.1007/s00216-025-06057-x.

## Introduction

Indoor air quality (IAQ) is influenced by different factors such as temperature, humidity, and a wide variety of pollutants. Indoors, pollutants such as particulate matter (PM), volatile organic compounds (VOCs), ozone, and molds are often encountered at higher concentrations than outdoors due to both indoor source contribution (cooking, cleaning, smoking, and resuspension) and indoor accumulation resulting from low ventilation rates [1, 2]. As people in high-income countries spend on average 85% of their time indoors, the overall exposure to air pollution and a person’s individual health and well-being is closely linked to IAQ [1, 3–5]. Symptoms of discomfort, adverse health effects, and increased absences from work or school, as well as degraded cognitive performances, have been linked to poor IAQ [6, 7].

Flame retardants (FRs) and plasticizers are used as functional additives in plastics, textiles, electronic equipment, furniture, and building materials [8]. FRs are added to reduce flammability in materials in order to comply with fire regulations [9]. Plasticizers improve compound-specific physicochemical characteristics of plastic polymers, such as flexibility, durability, and stretchability [10, 11]. On a global scale, around 9 million tons of plasticizers were sold in 2022 [12]. They are semi-volatile organic compounds (SVOCs), indicating that they are more stable than VOCs, difficult to degrade, and they persist for years in the environment [13]. Because they are usually dosed as additives to the polymer and not covalently bound to it, they can be subsequently released to the surrounding air, food, and water [11, 14]. This results in potential exposure through inhalation, ingestion, or dermal contact [15]. SVOCs with small molecular weight preferentially reside in the vapor phase, whereas those with large molecular weight accumulate in the particulate phase [16]. As FRs have a non-polar structure, they are prone to reside in non-polar organic matrices, such as lipid-rich animal tissue [17]. Indeed, organophosphate esters (OPEs), compounds used as FRs and plasticizers, were found in human urine, serum, milk, hair, and nails, suggesting their potential to bioaccumulate [9, 18].

Brominated FRs (BFRs) have been banned under Annex D of the Stockholm Convention on Persistent Organic Pollutants (POPs) due to their persistence, bioaccumulation, and toxicity (PBT) [19]. They have largely been replaced by OPEs, which are used as both FRs and plasticizers. Phthalate esters (PEs) and alternative plasticizers (APs), including adipates, terephthalates, citrates, trimellitates, and sebacates, are currently being used as plasticizers [20].

Certain FRs and plasticizers pose health risks, including endocrine and reproductive disruption, developmental effects, suspected carcinogenicity, associations with conditions related to asthma, and sick building syndrome [21]. OPEs are linked to bioaccumulation, persistence, and adverse effects on fertility, sperm quality, and the nervous system [21, 22], while PEs are suspected endocrine disruptors associated with allergic symptoms, asthma, and reproductive defects [23, 24]. The PEs bis(2-ethylhexyl) phthalate (DEHP), benzyl butyl phthalate (BBzP), di-n-butyl phthalate (DnBP), and di-iso-butyl phthalate (DiBP) are listed as Substances of Very High Concern (SVHC) under REACH, the Registration, Evaluation, Authorization and Restriction of Chemicals regulation implemented by the European Union. Accordingly, these phthalates are (among others) restricted in children’s toys in the USA [25, 26].

While APs are considered promising, less toxic alternatives, studies evaluating their leachability and toxicological impacts remain limited [17, 26]. The US Environmental Protection Agency (USEPA) uses the reference dose (RfD) and the oral slope factor (SFO) to assess non-carcinogenic and carcinogenic risks, respectively (see Table S1). Currently, the toxicity of PEs is attributed to their metabolites, leading to restrictions under Annex XVII of REACH [20]. Further research is needed to assess the safety of non-phthalate plasticizers.

Previous studies have performed analysis of OPEs, PEs, and/or APs in air samples on either the vapor or the particulate phase, often combining various techniques to account for both phases. For determinations of the vapor phase, polyurethane foam (PUF) is commonly used in either an active [10, 27] or passive [9, 28] sampling configuration. The particulate phase is often sampled using glass fiber filters (GFF) [10, 27, 29–31]. Collection on a cartridge with a quartz fiber filter (QFF) and PUF in tandem with cartridges was used for a smaller set of OPEs, PEs, and APs [27, 32, 33]. Apart from analyzing fewer compounds, the extraction method for QFF uses dichloromethane, which is more toxic than the n-hexane:acetone solvent used in this study. Additionally, extensive pre-cleaning is required on the QFF and PUF to avoid residual compounds, requiring more solvent and time.

A more recent development is the use of solid-phase extraction (SPE) membranes, cartridges, and solid-phase micro-extraction (SPME) to collect both phases simultaneously [34]. In the literature, compound analysis has been carried out by gas chromatography-mass spectrometry (MS), as well as liquid chromatography coupled to tandem mass spectrometry (LC-MS/MS), liquid chromatography-quadrupole-linear ion trap mass spectrometry (LC-QqLIT-MS), and quadrupole-Orbitrap high-resolution mass spectrometer (HRMS) [14, 35–38]. In general, LC is more suitable than GC for compounds that are thermally labile, non-volatile, and polar such as plasticizers without the need to significantly modify the sample [39].

The present study aims to develop a harmonized, analytical method for the determination of a wide range of legacy and emerging FRs and plasticizers in indoor air samples. Two previous methodologies were merged, extended, adapted, optimized, and validated for a larger number of contaminants [10, 40]. The main advantages of this new method are that SPE cartridges collect both the vapor and particulate phase simultaneously, they are small and robust, and compounds can be detected without pretreatment, thus reducing the material cost and sample handling time. Although the SPE setup is compatible with the extraction of other compound classes such as polycyclic aromatic hydrocarbons (PAHs), PAHs were not included in the current method development or analysis. They are mentioned here solely to highlight the broader potential of the approach.

## Materials and methods

### Chemicals and reagents

Sixteen OPEs, 13 PEs, 12 APs, and 21 labelled compounds used as internal standards (IS) were determined by means of this analytical method. Table S2 contains information on these compounds and their corresponding standards. Acetone and hexane solvents for organic trace analysis were obtained from J.T. Baker (Center Valley, PA, USA). Methanol and water solvents, ammonium acetate, and formic acid for trace analysis were purchased from Merck (Darmstadt, Germany). SPE cartridges (Isolute ENV +, 200 mg, 6 mL) were provided by Biotage (Uppsala, Sweden).

Special attention was paid to potential contamination from laboratory materials, given that certain lab equipment used for both sampling, extraction, and analysis are also made of plastic. This poses an additional challenge in assigning blanks. Adequate laboratory practices, such as avoiding plastic materials; working within a fume hood; pre-cleaning the glassware with ultrapure water, ethanol, and acetone; and heating at 380 °C, were maintained to avoid contamination, and a laboratory blank was included for each batch of samples.

### Sample extraction

Sample extraction was carried out according to Tao et al. [31]. The cartridges were spiked with 25 ng of the IS. Samples were eluted with 10 mL of n-hexane/acetone (1:1, v/v). After drying under a gentle stream of nitrogen until 1 mL, the sample was divided, using 500 µL for FR and plasticizer analyses and 500 µL for PAH analyses [41]. The 500 µL was further evaporated until dryness using nitrogen and reconstituted with 200 μL of methanol.

The method reported by Fernández-Arribas et al. [40] included 20 OPEs, 10 PEs, and 4 APs and was validated for the determination of plasticizers in a variety of matrices, including the particulate phase of air samples collected using filters. The new method was adapted to include an additional two PEs and eight APs in a sampling strategy using SPE cartridges, collecting both the particulate and gaseous phases of air samples.

For the analysis of plasticizers and FRs, online sample purification and analysis were performed with a Thermo Scientific TurboFlow™system, consisting of a triple quadrupole (QqQ) MS with a heated-electrospray ionization source (H-ESI), two LC quaternary pumps, and three LC columns, two of them for purification and the last one for separation. The TurboFlow™ purification columns used were Cyclone™-P (0.5 × 50 mm) and C18-XL (0.5 × 50 mm). Chromatographic separation was subsequently achieved using an analytical column Purosphere Star RP-18 (125 mm × 0.2 mm), with a particle size of 5 μm [35]. Instrumental conditions were adapted from Giulivo et al. [35] and are reported in Table S3.

To achieve the highest signal intensity, MS/MS conditions were optimized in spray positive mode for the new compounds: MEHP, DPHP, DMA, TEC, ATEC, DIPA, TBC, DBA, BTHC, and TOTM. Default values that were initially established for OPEs by Giulivo et al. [35] were used for positive voltage (3600 V), LC flow rate (5 μL/min), sheath and auxiliary gas (5 arbitrary units (Arb)), sweep gas (0 Arb), ion transfer tube temperature (320°C), and vaporizer temperature (50°C). Declustering potential (DP) and collision energy (CE), among other parameters, were optimized by infusing plasticizer standards directly into H-ESI, followed by MS detection in full scan mode. For each compound, two selective reaction monitoring (SRM) transitions were appointed for quantification and identification purposes.

Compounds were considered to be identified if both transitions (SRM1 and SRM2) (i) occurred at the same retention time (Rt) as the commercial standard; (ii) the signal to noise ratio was greater than 3; and (iii) if the peaks of both transitions had the same area ratio (± 15%) as that obtained through the commercial standards.

### Method evaluation

#### Quality parameters

An eight-point calibration curve in methanol comprising all 41 compounds was prepared to evaluate linearity. Analyte concentrations ranged from 0.1 to 1000 ng/mL, while maintaining the IS concentration at 50 ng/mL. Standard mixtures were prepared in methanol and stored at −20°C. Reproducibility was evaluated using the relative standard deviation (RSD) of five injections (*n*=5) on the same day (intra-day) and five injections (*n*=5) on consecutive days (inter-day) of a 50 ng/mL analyte and IS solution. The minimum analyte quantity that produced a signal to noise ratio (S/N) of 3 and 10 was used to calculate the instrumental limits of detection (iLODs) and instrumental limits of quantification (iLOQs), respectively.

Four replicas of cartridges were spiked at two different levels (*n*=8) to determine the analyte recovery of the developed method. The SPE cartridges were used directly from their commercial packages without any previous cleaning or conditioning step. OPEs were spiked at 20 ng (low level) and 100 ng (high level), while PEs and APs were spiked at 50 ng (low level) and 500 ng (high level) before extraction. Samples were eluted with 10 mL of n-hexane/acetone (1:1, v/v). After drying under a gentle stream of nitrogen, the sample was reconstituted in 500 µL of methanol. Twenty-five nanograms of IS mixture was added to the final solution. For each batch of samples, an SPE cartridge was added as blank, assessing the potential contamination due to the matrix and extraction procedure. The method limits of detection (mLODs) and method limits of quantification (mLOQs) for both high- and low-level spiked samples were calculated using four replicates of low-level spiked cartridges.

#### Storage and preservation tests

Storage tests were conducted to evaluate the stability of the compounds at three selected temperatures (23°C room temperature, 4 °C, and −20°C) and over a prolonged period of time (1 and 2 weeks, and 3 months). Triplicates of spiked, unpretreated SPE cartridges (20 ng of a native compound mix) were used to simulate sample shipping and storage conditions and evaluate compound behavior. The cartridges were wrapped in aluminum foil, placed within commercial heat-sealable silicon bags, provided by Kapak®, and stored under the aforementioned conditions until extraction, where 25 ng of an IS mix was added.

### Testing on indoor samples

Indoor air samples were collected in four offices at an urban environment in Barcelona (Spain), in May and June of 2023, in a case study approach. In each sampling site, one low-volume pump (Leland Legacy, SKC Inc., Eighty Four, PA) was deployed at an air flow rate of 4 L/min for 16 h: 2 days of 8-h sampling during working hours (9:00–17:00), corresponding to a total volume of 3.84 m^3^ per sample. One unpretreated SPE cartridge per location was used to collect both the gaseous and particulate phase (Fig. S1). The pumps operated inside soundproofed boxes to minimize the noise generated. In total, one valid sample was collected per office, as the real-world samples were only intended as validation of the method, not for the actual characterization of the indoor air concentrations.

Breakthrough of compounds in the sampling setup was tested by placing two SPE cartridges in tandem and spiking the first SPE with 20 ng of the IS mix. All samples were stored at −20°C and protected from light until analysis, which was performed within 1 week.

## Results and discussion

### Optimization of instrumental conditions

Two transitions for each of the new compounds, MEHP, DPHP, DMA, TEC, ATEC, DIPA, TBC, DBA, BTHC, and TOTM, were optimized. Transitions for the rest of the compounds were previously optimized [40]. Table 1 contains the instrumental parameters, including retention times (Rt), transitions (SRM_1_ and SRM_2_), declustering potential (DP), and collision energy (CE_1_ and CE_2_). As DiBP and DnBP coelute, the joint values are shown. The final experiment was designed using three retention time windows, from 0.5 to 7 min, from 7 to 21 min, and from 21 to 38 min, in order to improve the sensitivity. Table 1 also shows the intra-day and inter-day repeatability, and iLODs and iLOQs for each analyte. As for repeatability, the %RSD values were below 15% for both intra- and inter-day assays. The intra-day RSD values were expected to be lower than the inter-day values because measurements taken within the same day experience more consistent instrument conditions, reagent stability, and operator handling, whereas inter-day measurements are affected by variations in environmental factors, equipment recalibration, and sample degradation over time. The intra-day RSD was lower than the inter-day RSD for most compounds with the exception of TCEP and TNBP, where the values remained within the same range. Regarding sensitivity, for OPEs, PEs, and APs, iLOD values varied from 0.51 to 83.4, from 0.08 to 273, and from 0.10 to 16.8 pg, and the iLOQ values ranged from 1.45 to 278, from 0.26 to 910, and from 0.34 to 56.0 pg, respectively. If we compare these data with those obtained in the previously published method (Fernández-Arribas et al. 2024), in general, higher sensitivity for OPEs (0.01–1.36 pg) was previously observed, while the PEs-APs range is similar to the own APs range (0.02–18.5 pg). An increase in sensitivity was observed for two compounds, DMP and DEHA. Regarding the new compounds, sensitivity for new PEs and APs is in the same order. Our higher OPEs iLOD values could be explained by the fact that we are monitoring 41 compounds instead of the 34 compounds included in the previous study.

**Table 1:** Instrumental TFC-LC–MS/MS parameters: retention times (Rt), transitions (SRM1 and SRM2), declustering potential (DP), and collision energy (CE1 and CE2); repeatability and instrumental detection and quantification limits for each compound

	Rt (min)	SRM_1_/SRM_2_	DP (V)	CE_1_/CE_2_ (V)	Intraday (*n* = 5) (%RSD)	Interday (*n* = 5) (%RSD)	iLOD (pg)	iLOQ (pg)
OPEs								
TEP	4.5	183 → 99	36	19	0.7	1.5	0.52	1.74
		183 → 81		35				
TCEP	4.6	287 → 99	58	39	1.5	1.2	3.78	12.6
		287 → 224		16				
TCIPP	9.4	327 → 99	62	24	1.6	2.2	1.63	5.44
		327 → 250		10				
TDClPP	10.9	431 → 99	78	27	3.3	3.6	11.5	38.3
		431 → 320		16				
TPHP	11.3	327 → 152	83	39	1.0	1.0	1.18	3.95
		327 → 215		27				
TNBP	11.9	267 → 99	47	20	3.7	3.7	0.51	1.70
		267 → 210		8				
DCP	12.3	341 → 151	80	36	3.3	7.1	4.03	13.5
		341 → 228		27				
TBOEP	12.6	399 → 299	69	13	2.1	2.6	1.59	5.29
		399 → 199		17				
2IPPDPP	13.5	369 → 327	77	19	2.1	5.6	1.89	6.28
		369 → 152		39				
4IPPDPP	14	369 → 327	86	20	1.8	4.9	1.64	5.45
		369 → 152		39				
TmCP	14.4	369 → 165	54	59	1.4	4.5	0.82	2.72
		369 → 91		41				
EHDPP	15.1	363 → 251	66	15	1.1	4.5	9.86	32.9
		363 → 151		45				
B4IPPPP	16.6	411 → 152	87	44	1.7	8.4	2.08	6.94
		411 → 327		24				
IDPP	17.0	391 → 251	80	26	1.6	9.2	4.82	16.1
		391 → 152		53				
T2IPPP	17.1	453 → 327	87	29	1.7	7.48	1.01	3.36
		453 → 369		27				
TEHP	27.8	435 → 80	65	487	9.6	14.7	83.4	278
		435 → 99		102				
PEs								
DMP	4.6	195 → 163	30	32	1.9	3.1	0.80	2.66
		195 → 77		12				
DEP	8.5	223 → 149	30	30	4.5	3.1	0.09	0.31
		223 → 224		19				
** MEHP**	11.4	279 → 149	44	18	2.0	1.3	38.4	128
		279 → 121		35				
** DPHP**	11.5	319 → 225	47	13	8.4	4.2	0.71	2.35
		319 → 77		34				
DiBP-DnBP^a^	12.3–12.5	279 → 149	30	18	1.9	1.9	27.6	91.8
		279 → 121		36				
BBzP	12.7	313 → 91	34	23	2.1	7.6	1.02	3.39
		313 → 149		23				
DCHP	15.1	331 → 149	33	25	2.3	4.7	0.73	2.43
		331 → 121		45				
DHexP	17.4	335 → 149	41	18	3.1	3.3	0.17	0.57
		335 → 121		40				
DEHP	23.2	391 → 149	46	23	2.8	5.4	0.08	0.26
		391 → 167		13				
DnOP	25.8	391 → 149	60	19	2.3	1.3	26.1	87.0
		391 → 121		50				
DiNP	26.3	419 → 149	54	23	10.4	1.3	273	910
		419 → 121		47				
DiDP	28.7	447 → 149	57	23	7.1	6.9	183	609
		447 → 121		49				
APs								
** DMA**	4.5	175 → 111	35	13	1.7	7.4	11.7	38.8
		175 → 115		15				
** TEC**	4.6	277 → 111	50	28	2.9	5.5	1.82	6.07
		277 → 69		43				
** ATEC**	5.1	319 → 139	47	25	2.9	14.5	0.31	1.05
		319 → 115		30				
** DIPA**	10.2	231 → 111	31	19	9.8	14.6	0.10	0.34
		231 → 101		22				
** TBC**	12.4	361 → 129	54	22	5.2	9.3	0.29	0.97
		361 → 111		30				
** DBA**	12.6	259 → 111	45	27	5.8	10.8	6.32	21.1
		259 → 101		21				
ATBC	13.8	403 → 129	49	27	5.6	4.2	0.60	2.01
		403 → 139		26				
** DEHA**	23.2	371 → 129	45	27	3.9	3.4	10.9	36.5
		371 → 111		27				
** BTHC**	25.4	515 → 129	67	30	2.1	4.7	3.18	10.6
		515 → 139		30				
DINA	27.5	399 → 111	48	22	4.3	8.4	12.3	41.1
		399 → 101		26				
DINCH	29.1	425 → 127	49	13	3.5	9.6	16.8	56.0
		425 → 109		47				
** TOTM**	33.9	547 → 305	71	21	4.9	8.2	16.0	53.3
		547 → 193		42				

Details of compound acronyms can be found in Table S2 of the Supplementary Information

In bold: new compounds added to the previous method, and for which analysis is optimized and validated

^a^Chromatographic resolution between DiBP and DnBP was insufficient; thus, both isomers were integrated and quantified together

### Method evaluation

#### Quality parameters

Analytical parameters of the developed method, including recoveries, reproducibility, mLODs, and mLOQs, are summarized in Table 2. Recoveries between 40 and 120% and reproducibility (%RSD) below 30% were deemed acceptable for method application to environmental real-world samples. Recovery tests were performed at low (*n* = 3): 20 ng (OPEs) or 50 ng (PEs and APs) and high concentrations (*n* = 3): 100 ng (OPEs) or 500 ng (PEs and APs). This analytical method provides low-level recoveries between 56 and 104%, 50–114%, and 59–113% for OPEs, PEs and APs, respectively. Similarly, high-level recoveries from 60 to 136%, 73 to 116%, and 55 to 119% were obtained for OPEs, PEs, and APs, respectively, with reproducibility always below 21%.

For certain analytes, no acceptable recoveries (lower than 40% or exceeding 120%) were obtained: TEP, DiBP-DnBP, DMA, DIPA, TBC, ATBC, DINA, and TOTM, resulting in the inability to monitor these compounds using the proposed method. TEP showed insufficient recovery values, which could have been caused by its high volatility; similar results have been observed using other published methods, with filters as a sampling device [6]. In the case of DiBP-DnBP, the poor recoveries are probably due to the high values obtained for these compounds in the laboratory blanks, well above the levels spiked for recovery tests, which can be reduced by pre-cleaning the cartridges in advance. Furthermore, the coelution of both DiBP and DnBP presents a challenge for peak integration that can be resolved using gas chromatography. DMA, DIPA, TBC, ATBC, DINA, and TOTM are all alternative plasticizers which are considered to be less persistent in the environment compared to legacy plasticizers and thus be less stable [26]. Even though the recoveries for B4IPPPP and T2IPPP exceed 130% for the high spike, they are included in the method because the low spike is more relevant for environmental samples. These elevated recoveries could be attributed to residual impurities from the plastic SPE cartridges, which may cause ionization enhancement during HPLC–MS/MS analysis.

Regarding sensitivity, mLOD for all analytes varied between 0.02–1.94 ng/m^3^ (low spike) and 0.04–20 ng/m^3^ (high spike). mLOQ for OPEs varied between 0.06–1.10 ng/m^3^ and 0.20–2.97 ng/m^3^ for low and high spiked concentrations, respectively. mLOQ values in PEs were 0.06–6.46 ng/m^3^ (low spike) and 0.12–66.5 ng/m^3^ (high spike). APs mLOQ values varied between 0.12–2.12 ng/m^3^ (low spike) and 1.40–4.90 ng/m^3^ (high spike). In general, values of the same magnitude were obtained, except for DEP showing higher mLOQ values. Because the low spike level is intended to reflect the method’s sensitivity at environmentally relevant concentrations, the mLOD and mLOQ values determined from the low spike are considered the valid indicators of method performance. For illustration purposes, Fig. S2 shows the LC–MS/MS chromatograms obtained for the 33 quantifiable analytes.

Comparing the analytical parameters of the proposed method to the established method for quartz fiber filters [40], the proposed method showed similar recoveries and sensitivity for OPEs, while the eight common PEs and APs showed higher recoveries, with six also showing higher sensitivity (Table 2). Additionally, the proposed method enabled the detection of two more PEs and four more APs. Finally, the proposed method captures both the particulate and gas phases in a single sample, offering a compact, streamlined approach that reduces handling and avoids phase separation. However, unlike the QFF method, it does not allow for phase-specific or PM_2.5_-targeted analysis, which may still be required in studies focused on particle-phase resolution. A direct comparison, using both the adapted and established methods in the same environment, would clarify differences in method performance. When compared with other published studies (see Table S4), the reported method performance for all three compound groups lies within the same range. For OPEs, the higher upper mLOD observed is attributed to the inclusion of a greater number of target analytes. The newly included compounds were selected due to their growing use as alternative plasticizers and recent environmental detection, supporting monitoring of emerging contaminants.

**Table 2:** Comparison of the analytical parameters (recovery, mLOD, and mLOQ) between the established and adapted method

		Established method		Adapted method		Established method		Adapted method	
		Recovery	%RSD	Recovery	%RSD	mLOD (pg/m^3^)	mLOQ (pg/m^3^)	mLOD (ng/m^3^)	mLOQ (ng/m^3^)
OPEs									
TCEP	Low	64	3	77	4	0.01	0.04	0.07	0.22
	High	61	0	85	4	0.04	0.14	0.18	0.59
TCIPP	Low	64	4	69	6	0.02	0.08	0.04	0.12
	High	57	10	77	3	0.06	0.20	0.13	0.44
TDCIPP	Low	83	3	65	7	0.03	0.11	0.10	0.33
	High	79	2	83	2	0.06	0.21	0.08	0.26
TPHP	Low	83	2	77	9	0.00	0.01	0.04	0.13
	High	77	3	85	1	0.01	0.03	0.11	0.35
TNBP	Low	85	9	56	7	0.002	0.01	0.02	0.06
	High	69	12	60	5	0.01	0.03	0.06	0.20
DCP	Low	117	1	97	11	0.01	0.02	0.12	0.39
	High	98	1	84	7	0.02	0.05	0.19	0.64
TBOEP	Low	64	2	77	8	0.00	0.01	0.04	0.12
	High	62	3	87	1	0.01	0.05	0.19	0.62
2IPPDPP	Low	82	2	83	11	0.00	0.01	0.07	0.22
	High	88	10	89	1	0.01	0.02	0.37	1.24
4IPPDPP	Low	88	4	87	14	0.00	0.01	0.07	0.24
	High	101	12	100	2	0.01	0.02	0.46	1.54
TMCP	Low	98	2	84	8	0.00	0.01	0.14	0.48
	High	89	6	117	2	0.01	0.02	0.69	2.28
EHDPP	Low	83	3	84	8	0.02	0.07	0.16	0.53
	High	85	11	102	3	0.07	0.22	0.65	2.17
B4IPPPP	Low	63	5	83	14	0.00	0.01	0.25	0.83
	High	74	10	136	5	0.01	0.02	0.58	1.94
IDPP	Low	40	11	82	12	0.05	0.17	0.12	0.41
	High	49	18	113	3	0.16	0.52	0.16	0.53
T2IPPP	Low	16	7	76	21	0.01	0.05	0.03	0.11
	High	19	16	130	7	0.04	0.12	0.11	0.37
TEHP	Low	86	9	104	15	0.08	0.27	0.33	1.10
	High	75	1	98	9	0.17	0.55	0.89	2.97
PEs									
DMP	Low	20	57	96	4	0.05	0.18	0.05	0.16
	High	11	59	73	3	0.10	0.33	0.09	0.31
DEP	Low	143	23	91	1	0.03	0.10	1.94	6.46
	High	78	29	86	1	0.12	0.40	20.0	66.5
** MEHP**	Low			67	9			0.28	0.94
	High			82	2			0.28	0.93
** DPHP**	Low			50	4			0.04	0.12
	High			90	3			0.10	0.32
BBzP	Low	56	8	61	18	0.01	0.03	0.12	0.41
	High	59	5	73	4	0.02	0.05	0.37	1.22
DCHP	Low	43	2	74	5	0.001	0.004	0.03	0.11
	High	39	7	86	1	0.004	0.01	0.03	0.10
DHexP	Low	65	7	70	9	0.01	0.02	0.02	0.06
	High	55	4	82	2	0.01	0.03	0.04	0.14
DEHP	Low	36	26	86	3	0.02	0.06	0.93	3.09
	High	58	10	105	2	0.06	0.20	3.63	12.09
DnOP	Low	64	7	114	2	0.07	0.22	0.66	2.20
	High	60	9	110	2	0.07	0.22	3.64	12.13
DiNP	Low	53	4	87	12	0.93	3.10	1.71	5.71
	High	76	5	114	4	0.78	2.60	1.93	6.43
DiDP	Low	8	74	103	16	0.38	1.26	0.79	2.63
	High	28	13	116	5	0.30	1.00	1.49	4.98
APs									
** TEC**	Low			106	8			0.11	0.38
	High			113	3			0.65	2.17
** ATEC**	Low			113	2			0.07	0.23
	High			119	4			0.70	2.34
** DBA**	Low			110	6			0.37	1.25
	High			101	13			1.47	4.90
DEHA	Low	57	9	81	5	0.10	0.35	1.22	4.07
	High	60	11	80	5	0.09	0.31	1.76	5.86
** BTHC**	Low			59	19			0.23	0.78
	High			55	2			0.47	1.56
DINA	Low	80	2	95	6	0.10	0.33	0.42	1.40
	High	82	9	106	4	0.16	0.52	0.81	2.69
DINCH	Low	32	4	105	6	0.08	0.26	0.70	2.34
	High	33	7	99	5	0.14	0.47	1.10	3.68

Lastly, for the validation of the sampling method with the SPE Isolute ENV + cartridges, a breakthrough test was performed. To replicate the actual sampling conditions, an air flow of 4 L/min was used for 8 h. The IS concentrations detected in the tandem cartridges were below 0.25% of the concentrations detected in the spiked cartridge, indicating that the setup does not result in breakthrough.

#### Storage and preservation tests

Various analytical methods have been developed to evaluate different pollutants in ambient air. However, the stability of these pollutants from the time of sampling to laboratory analysis remains insufficiently studied. In this study, storage tests were conducted to assess the preservation of the compounds under various temperature conditions (23 °C room temperature, 4 °C, and − 20 °C) over different time intervals (1 week, 2 weeks, and 3 months). The storage of air samples in aluminum foil and airtight polyethylene bags at low temperature (− 20 °C) has previously shown to be advantageous compared to storage in fragile glassware [14]. To date, no study has assessed whether the storage and preservation methods used prevented the degradation of the analytes of interest, specifically plasticizers and FRs.

**Fig. 1:**
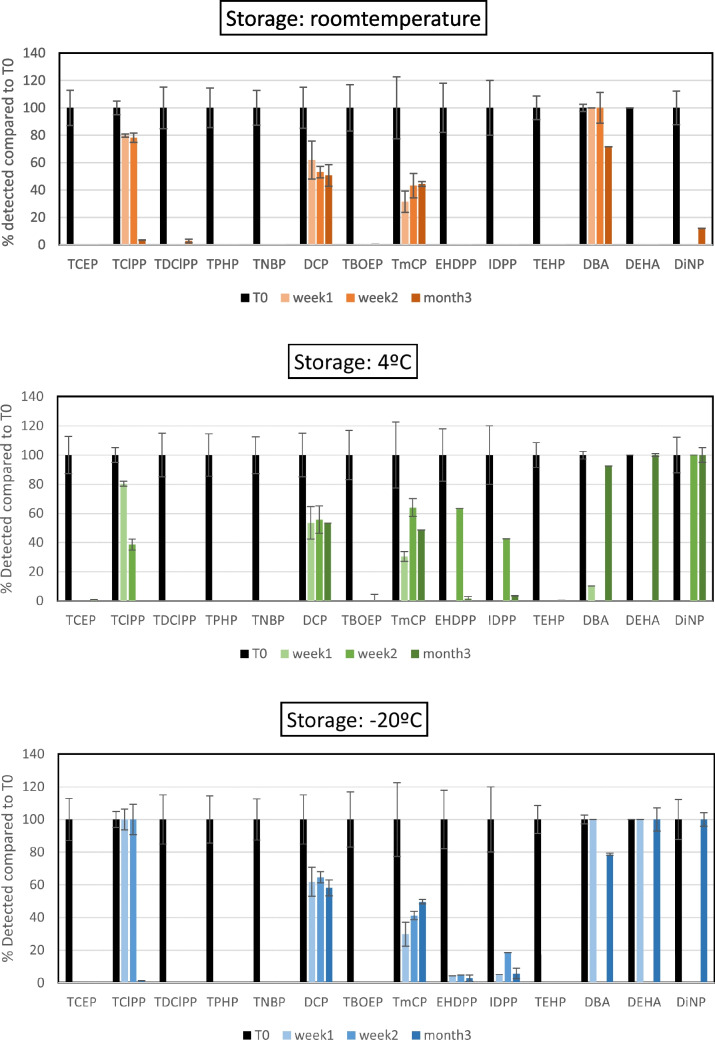
Percentage of detected plasticizers compared to initial conditions (T0). Top–bottom: storage at room temperature, 4* °C*, and − 20* °C*. T0 (black): extracted on the day of the spike (control); Tw1, Tw2, Tm3 extracted respectively, 1 week, 2 weeks, and 3 months after spiking

Of the 33 compounds for which the analytical method has been developed, 14 present stability problems. Figure 1 illustrates the percentage of each compound detected relative to the initial conditions (T0) for those compounds that were detected in less than 80% of the initial concentrations (T0) after 1 and/or 2 weeks of preservation. Most unstable compounds are OPEs that show two main trends: Certain compounds (TCEP, TDCIPP, TPHP, TNBP, TBOEP, and TEHP) were only detected immediately after spiking and were thus lost during storage, indicating their unsuitability for long-term storage. This instability is likely due to a combination of factors, including volatility, adsorption to cartridge or vial surfaces, and possible chemical degradation or hydrolysis. Other compounds (DCP, TmCP, EHDPP, and IDPP) were still detected after certain time intervals up to 65% of the initial concentrations. Thirdly, TCIPP, DBA, DEHA, and DiNP show 100% detection relative to their initial concentrations (T0) under specific storage conditions; however, under other conditions, they are not detected at all. To monitor these last two groups in real-life samples, extractions are recommended to be performed immediately after sampling. For DCP, TmCP, EHDPP, and IDPP, if samples are stored before extraction, it must be considered that their detected concentrations may be underestimated. After 2 weeks of storage, the other 19 compounds retained at least 80% of their initial concentration when stored at 4 °C (see Fig. S4 and Fig. S5). Overall, the lowest concentrations tend to be detected after 3 months; however, only DPHP, DHexP, DiDP, and ATEC fell below this threshold. OPEs showed the least fluctuation across temperatures, with smaller standard deviations than PEs and APs. B4IPPP and DPHP were below 80% at both room temperature and − 20 °C.

As a result, it was concluded that extractions should be performed within 2 weeks of sampling, and storing the samples at least at 4 °C. To ensure accuracy, the addition of a sampling internal standard (different to the analytical internal standard) could be considered relevant to avoid underestimation of compound concentrations due to losses by instability.

#### Levels of plasticizers and flame retardants in indoor air

**Fig. 2:**
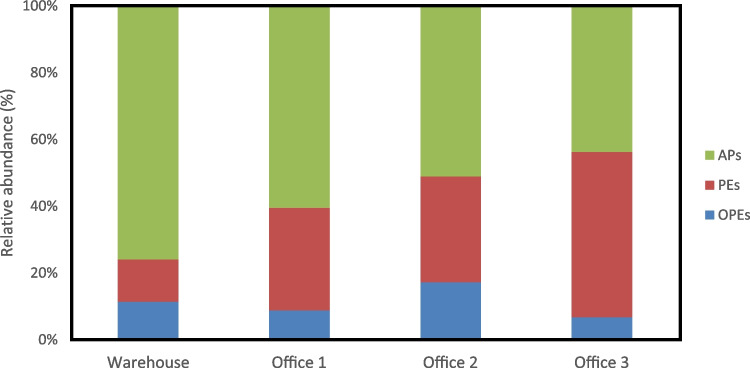
Relative abundance (%) of OPEs, PEs, and APs at each location

All analyzed samples contained detectable levels of plasticizers ranging from 0.00518 to 28.9 ng/m^3^ (Table S5), with compounds from all three target families (OPEs, PEs, and APs) present. As shown in Fig. 2, Offices 1 and 2 shared similar compound profiles, dominated by APs and containing comparable levels of PEs. Office 3 exhibited a slightly more balanced distribution between APs and PEs, with a lower presence of OPEs. The warehouse showed the most distinct profile, with APs comprising nearly 80% of detected compounds. These findings underscore the relevance of including APs in indoor air monitoring. The results also highlight the utility of the developed method for simultaneously analyzing both well-established and emerging plasticizers.

**Fig. 3:**
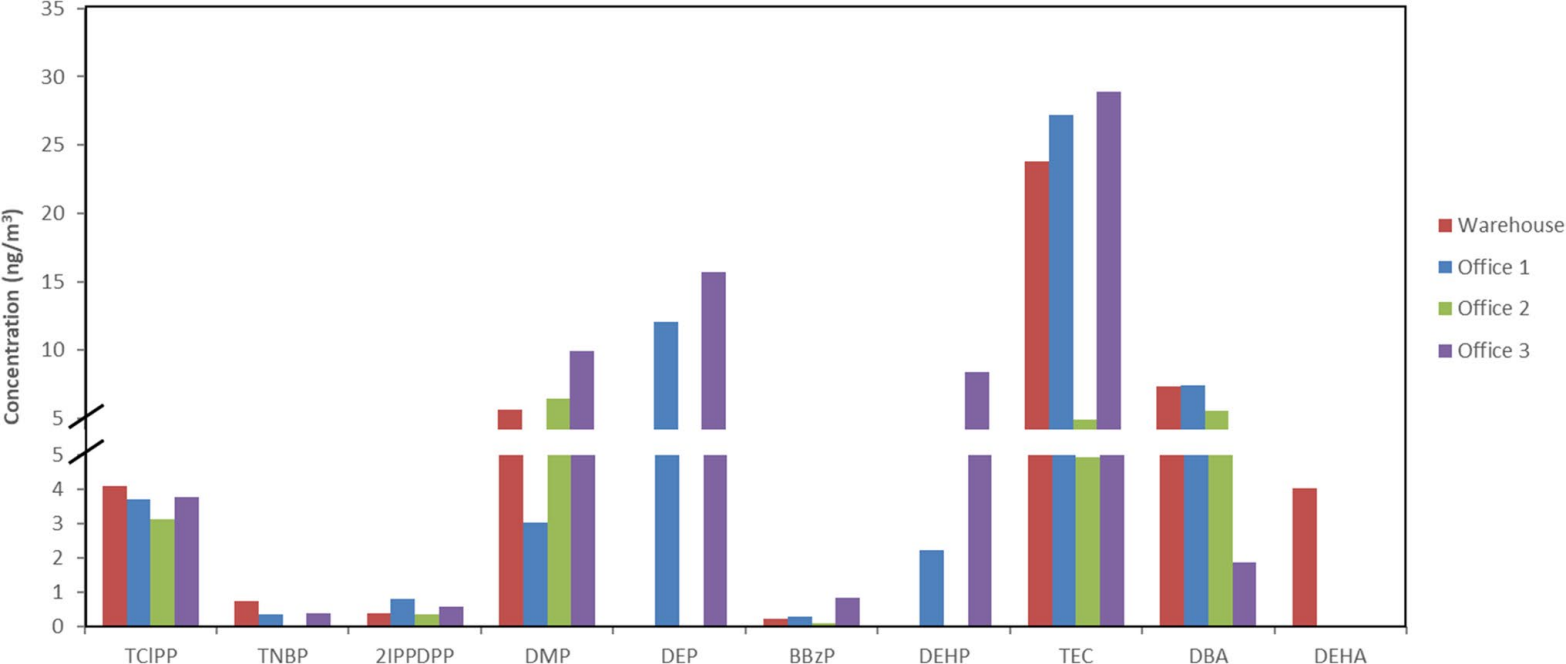
Concentration levels (ng/m^3^) of detected OPEs, PEs, and APs in indoor air samples

As samples were collected and analyzed immediately, all 33 targeted compounds in the developed methodology were determined. Out of 33 analyzed compounds, 9 OPEs, 6 PEs, and 6 APs were detected. Figure 3 shows the levels detected and quantified in all four samples. TCIPP, TNBP, 2IPPDPP, DMP, BBZP, TEC, and DBA were detected at all sampling locations. TEC was the most abundant compound in three locations, with significantly higher concentrations than other detected analytes. In contrast, Office 2 exhibited overall lower concentrations, with TEC, DBA, and DMP present in similar amounts. Differences between locations may be linked to variations in ventilation, equipment, and occupancy. PEs and APs that were detected occurred within the lower range of previously observed concentrations [27, 32, 33]. Notably, 2IPPDPP was detected despite its rare inclusion in indoor monitoring studies [9, 10, 30, 32], and this study is the first to report TEC and TBC in air samples rather than dust [38].

## Conclusions

An adapted method using TFC-LC–MS/MS was developed and evaluated for the analysis of 33 FRs and plasticizers in the vapor and particulate phase, including 6 newly added plasticizers, and applied to indoor air samples. Recoveries, reproducibility, mLODs and mLOQs were calculated to evaluate the applicability of the developed methodology. Using SPE cartridges resulted in a general improvement of recoveries compared to the previously established method which used filters (ranging from 56–136% for OPEs, 50–116% for PEs, and 55–119% for APs), with %RSD below 21%. The sensitivity was evaluated, calculating mLODs and mLOQs: mLODs ranged from 0.02–0.33, 0.02–1.94, and 0.04–1.05 ng/m^3^ for low spiked OPEs, PEs, and APs, respectively, and mLOQs varied between 0.05–0.89, 0.04–19.95, and 0.12–4.07 ng/m^3^ for high spiked OPEs, PEs, and APs, respectively. Including a sampling internal standard was necessary to accurately analyze real-world samples and prevent underestimation of compound concentrations. Preservation tests showed that cartridge samples can be stored for at least 2 weeks at preferably 4 °C in order to maintain analyte integrity.

Lastly, the applicability of the method was evaluated using real-world samples. FRs and plasticizers were detected at levels comparable to the literature. Due to the banning of legacy plasticizers and FRs with hazardous effects, more APs are being manufactured and thus making them more prevalent in the environment. Because of its recent emergence, there is limited research available on APs. Their detection in the present study and their ongoing increase in usage suggest that the proposed methodology will be valuable in future studies.

## Supplementary Information

Below is the link to the electronic supplementary material.

Supplementary file1 (DOCX 1.20 MB)

## Data Availability

The data supporting the findings of this study are included in the article and its supplementary information files. Additional datasets generated and/or analyzed during the current study are available from the corresponding author upon reasonable request.
