# Supplementary material for: Development of a strategy for sampling, preservation, and analysis of classical and emerging flame retardants and plasticizers in air samples
Source: Anal Bioanal Chem. 2025 Sep 2;417(23):5335–47. doi: 10.1007/s00216-025-06057-x (PMC12432039; doi:10.1007/s00216-025-06057-x)
Supplement: Supplementary file 1 — Supplementary file1 (DOCX 1.20 MB) [file 216_2025_6057_MOESM1_ESM.docx]

Supplementary information

**Development of a strategy for sampling, preservation and analysis of classical and emerging flame retardants and plasticizers in air samples**

Judith Desmet, Maria A. Aretaki, Mar Viana, Ethel Eljarrat*

**Table S1** RfD and SFO values for OPEs, PEs and APs.

|  | RfD (ng/Kg bw/day) | SFO (mg/kg bw/day) |
| --- | --- | --- |
| OPEs |  |  |
| TCEP | 7000 | 0.02 |
| TClPP | 10000 | - |
| TDClPP | 20000 | - |
| TNBP | 10000 | 0.009 |
| TCP | 20000 | - |
| TEHP | 100000 | 0.0032 |
| TMP | 10000 | 0.02 |
| EHDPP^a^ | 600 | - |
| TPHP^b^ | 7000 | - |
| PEs |  |  |
| DEP | 800000 | - |
| DnBP | 100000 | - |
| BBzP | 200000 | 0.0019 |
| DEHP | 20000 | 0.014 |
| DnOP | 10000 | - |
| APs |  |  |
| DEHA | - | 0.0012 |

[1], a [2], b [3]

bw: body weight

**
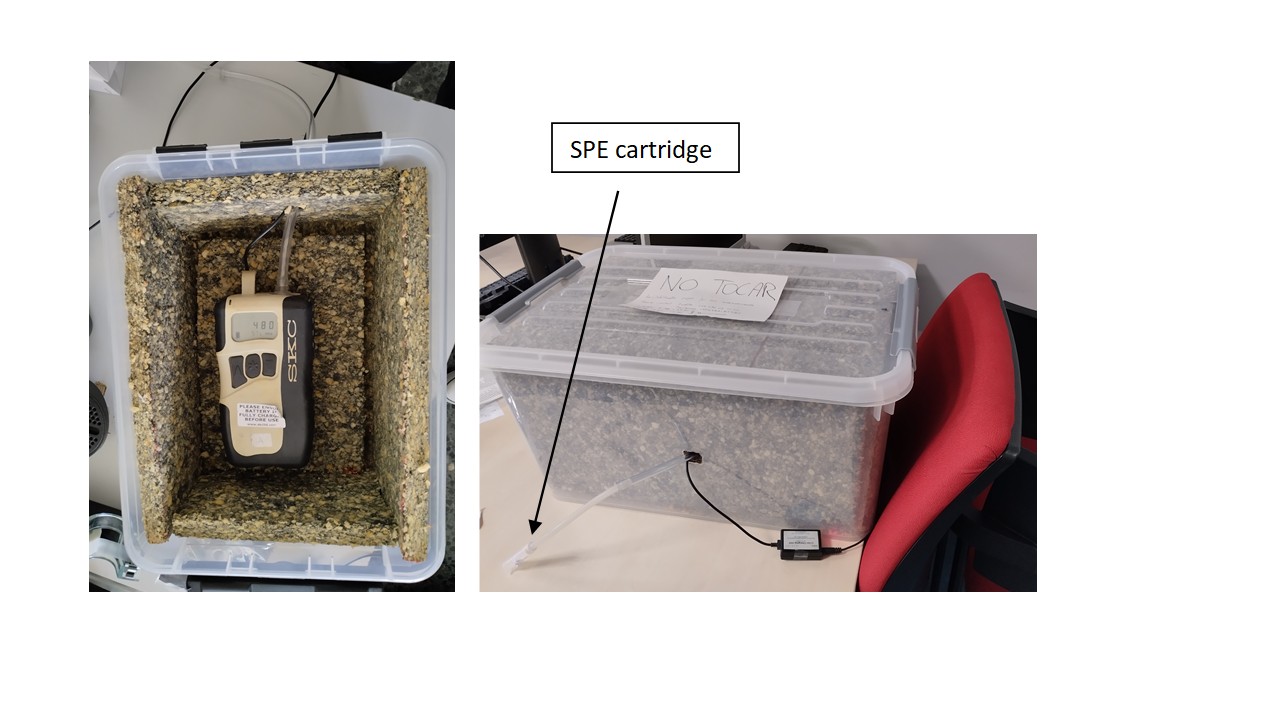
**

SPE cartridge

**Figure S1** Experimental setup: placement of pumps inside soundproof boxes on top of office desks.

**Table S2** Characteristics of standards and internal standards (IS) of FRs and plasticizers used in this study.

| Abbreviation | Full name | Molecular structure | Molecular weight (g/mol) | CAS number | provider | IS |
| --- | --- | --- | --- | --- | --- | --- |
| OPEs |  |  |  |  |  |  |
| TEP | triethyl phosphate | C_6_H_15_O_4_P | 182.15 | 78-40-0 | M | d_15_-TEP |
| TCEP | tris(2-chloroethyl) phosphate | C_6_H_12_Cl_3_O_4_P | 285.48 | 115-96-8 | SCB | d_12_-TCEP |
| TClPP | tris(2-chloroisopropyl) phosphate | C_9_H_18_Cl_3_O_4_P | 327.57 | 13674-84-5 | SCB | d_21_-TPrP |
| TDClPP | tris(1,3-dichloro-2-propyl) phosphate | C_9_H_15_Cl_6_O_4_P | 430.89 | 13674-87-8 | M | d_15_-TDClPP |
| TPHP | triphenyl phosphate | C_18_H_15_O_4_P | 326.28 | 115-86-6 | M | d_15_-TPHP |
| TNBP | tri-n-butyl phosphate | C_12_H_27_O_4_P | 266.32 | 126-73-8 | M | d_27_-TNBP |
| DCP | diphenylcresyl phosphate | C_19_H_17_O_4_P | 340.31 | 26444-49-5 | AS | d_27_-TNBP |
| TBOEP | tris(2-butoxyethyl) phosphate | C_18_H_39_O_7_P | 398.47 | 78-51-3 | SCB | ^13^C_2_-TBOEP |
| 2IPPDPP | 2-isopropylphenyl diphenyl phosphate | C_21_H_21_O_4_P | 368.36 | 64532-94-1 | TRC | ^13^C_2_-TBOEP |
| 4IPPDPP | 4-isopropylphenyl diphenyl phosphate | C_21_H_21_O_4_P | 368.36 | 55864-04-5 | TRC | ^13^C_2_-TBOEP |
| TmCP | tricresyl phosphate | C_21_H_21_O_4_P | 368.36 | 1330-78-5 | ES | ^13^C_2_-TBOEP |
| EHDPP | 2-ethylhexyldiphenyl phosphate | C_20_H_27_O_4_P | 362.40 | 1241-94-7 | AS | ^13^C_2_-TBOEP |
| B4IPPPP | bis(4-isopropylphenyl) phenyl phosphate | C_24_H_27_O_4_P | 410.40 | 55864-07-8 | WL | ^13^C_2_-TBOEP |
| IDPP | isodecyldiphenyl phosphate | C_22_H_31_O_4_P | 390.50 | 29761-21-5 | AS | ^13^C_2_-TBOEP |
| T2IPPP | tris(2-isopropylphenyl) phosphate | C_27_H_33_O_4_P | 452.50 | 64532-95-2 | AS | ^13^C_2_-TBOEP |
| TEHP | tris(2-ethylhexyl) phosphate | C_24_H_51_O_4_P | 434.63 | 78-42-2 | SCB | d_51_-TEHP |

**Table S2** (continued)

| PEs |  |  |  |  | |  |  |
| --- | --- | --- | --- | --- | --- | --- | --- |
| DMP | dimethyl phthalate | C_10_H_10_O_4_ | 194.18 | 131-11-3 | AS | | d_4_-DMP |
| DEP | diethyl phthalate | C_12_H_14_O_4_ | 222.24 | 84-66-2 | AS | | d_4_-DEP |
| **MEHP** | monoethylhexyl phthalate | C_16_H_22_O_4_ | 278.34 | 4376-20-9 | AS | | d_4_-MEHP |
| **DPHP** | diphenyl phthalate | C_28_H_46_O_4_ | 446.70 | 53306-54-0 | W | | d_4_-MEHP |
| DiBP | diisobutyl phthalate | C_16_H_22_O_4_ | 278.35 | 84-69-5 | AS | | d_4_-DiBP |
| BBzP | n-butyl benzyl phthalate | C_19_H_20_O_4_ | 312.36 | 85-68-7 | AS | | d_4_-DnBP |
| DnBP | di-n-butyl phthalate | C_16_H_22_O_4_ | 278.34 | 84-74-2 | AS | | d_4_-BBzP |
| DCHP | di-cyclohexyl phthalate | C_20_H_26_O_4_ | 330.42 | 84-61-7 | AS | | d_4_-DCHP |
| DHexP | dihexyl phthalate | C_20_H_30_O_4_ | 334.45 | 84-75-3 | AS | | d_4_-DHexP |
| DEHP | di(2-ethyl hexyl) phthalate | C_24_H_38_O_4_ | 390.56 | 117-81-7 | AS | | d_4_-DEHP |
| DnOP | di-n-octyl phthalate | C_24_H_38_O_4_ | 390.56 | 117-84-0 | AS | | d_4_-DnOP |
| DiNP | di-iso-nonyl phthalate | C_26_H_42_O_4_ | 418.62 | 28553-12-0 | AS | | d_4_-DnOP |
| DiDP | di-iso-decyl phthalate | C_28_H_46_O_4_ | 446.67 | 26761-40-0 | AS | | d_4_-DnOP |
| APs |  |  |  |  |  | |  |
| **DMA** | dimethyl adipate | C_8_H_14_O_4_ | 174.19 | 627-93-0 | ES | | d_4_-DMP |
| **TEC** | triethyl citrate | C_12_H_20_O_7_ | 276.29 | 77-93-0 | ES | | d_4_-DMP |
| **ATEC** | acetyl triethyl citrate | C_14_H_22_O_8_ | 318.32 | 77-89-4 | ES | | d_4_-DMP |
| **DIPA** | diisopropyl adipate | C_12_H_22_O_4_ | 230.30 | 6938-94-9 | W | | d_3_-ATBC |
| **TBC** | tributyl citrate | C_19_H_32_O_7_ | 360.45 | 77-94-1 | ES | | d_3_-ATBC |
| **DBA** | di-n-butyl adipate | C_14_H_26_O_4_ | 258.35 | 105-99-7 | ES | | d_3_-ATBC |
| ATBC | acetyl tributyl citrate | C_20_H_34_O_8_ | 402.48 | 77-90-7 | ES | | d_3_-ATBC |
| DEHA | bis(2-ethylhexyl) adipate | C_22_H_42_O_4_ | 370.57 | 103-23-1 | AS | | d_8_-DEHA |
| **BTHC** | butyryl trihexyl citrate | C_28_H_50_O_8_ | 514.70 | 82469-79-2 | ES | | d_4_-DnOP |
| DINA | diisononyl adipate | C_24_H_46_O_4_ | 398.60 | 33703-08-1 | ES | | d_4_-DnOP |
| DINCH | 1,2-cyclohexanedicarboxylic acid, bis-isononyl ester | C_26_H_48_O_4_ | 424.67 | 166412-78-8 | ES | | d_4_-DnOP |
| **TOTM** | tri(2-ethylhexyl) trimellitate | C_33_H_54_O_6_ | 546.78 | 3319-31-1 | ES | | d_4_-DnOP |

**Table S2** (continued)

| IS for quantification | |  |  |  |  |  |
| --- | --- | --- | --- | --- | --- | --- |
| d_15_-TEP | - | C_6_D_15_O_4_P | 197.25 | 135942-11-9 | LGC | - |
| d_12_-TCEP | - | C_6_D_12_ClO_4_P | 297.56 | 1276500-47-0 | LGC | - |
| d_21_-TPrP | - | C_9_D_21_O_4_P | 245.36 | 1219794-92-9 | LGC | - |
| d_15_-TDClPP | - | C_9_D_15_Cl_6_O_4_P | 446.00 | 1447569-77-8 | LGC | - |
| d_15_-TPHP | - | C_18_D_15_O_4_P | 341.38 | 1173020-30-8 | LGC | - |
| d_27_-TNBP | - | C_12_D_27_O_4_P | 293.48 | 61196-26-7 | LGC | - |
| ^13^C_2_-TBOEP | - | C_18_H_39_O_7_P | 398.50 | - | WL | - |
| d_51_-TEHP | - | C_24_D_51_O_4_P | 485.95 | 1259188-37-8 | LGC | - |
| d_4_-DMP | - | C_10_H_6_D_4_O_4_ | 198.21 | 93951-89-4 | AS | - |
| d_4_-DEP | - | C_12_H_10_D_4_O_4_ | 226.26 | 93952-12-6 | AS | - |
| d_4_-MEHP | - | C_16_H_18_D_4_O_4_ | 282.37 | 1276197-22-8 | CH | - |
| d_4_-DiBP | - | C_16_H_18_D_4_O_4_ | 282.37 | 358730-88-8 | AS | - |
| d_4_-BBzP | - | C_19_H_16_D_4_O_4_ | 316.38 | 93951-88-3 | ASS | - |
| d_4_-DnBP | - | C_16_H_18_D_4_O_4_ | 282.37 | 93952-11-5 | AS | - |
| d_4_-DCHP | - | C_20_H_22_D_4_O_4_ | 334.44 | 358731-25-6 | AS | - |
| d_4_-DHexP | - | C_20_H_26_D_4_O_4_ | 338.48 | 1015854-55-3 | AS | - |
| d_4_-DEHP | - | C_24_H_34_D_4_O_4_ | 394.58 | 93951-87-2 | ES | - |
| d_4_-DnOP | - | C_24_H_34_D_4_O_4_ | 394.58 | 93952-13-7 | AS | - |
| d_4_-DiNP | - | C_26_H_38_D_4_O_4_ | 422.64 | 1202865-43-7 | ES | - |
| d_3_-ATBC | - | C_20_H_31_D_3_O_8_ | 405.50 | 1794753-49-3 | CDN | - |
| d_8_-DEHA | - | C_22_H_34_D_8_O_4_ | 378.62 | 1214718-98-5 | CH | - |

In bold: new compounds that were added to the previously established method [4], and whose analysis was optimised and validated. M: Merck (Darmstadt, Germany), SCB: Santa Cruz Biotechnology (SantaCruz, CA, USA), AS: AccuStandard (New Haven, CT, USA), TRC: Toronto Research Chemicals (Toronto, Canada), ES: Dr.Ehrenstorfer GmbH (Augsburg, Germany), WL: Wellington Laboratories Inc. (Guelph, ON, Canada), W: WAKO (Osaka, Japan), LGC (Teddington, UK), CH: Chiron (Trondheim, Norway), CDN Isotopes Inc (Pointe-Claire, Canada), ES: Dr.Ehrenstorfer GmbH (Augsburg, Germany).

**Table S3** TFC-LC instrumental conditions for purification (pump 1) and chromatographic separation (pump 2) of analytes.

|  | | | **Pump 1: Load Pump (Turboflow)** | | | | | | **Pump 2: Elute Pump (Chromatographic)** | | | | | |
| --- | --- | --- | --- | --- | --- | --- | --- | --- | --- | --- | --- | --- | --- | --- |
|  |  |  | Injection volume: 20 μL | | | | | |  | | | | | |
|  |  |  | Purification columns: CycloneTM-P (0.5x50mm)  C18-XL (0.5x50mm) | | | | | | Analytical column: Purosphere STAR RP-18 (125mmx0.2mm) | | | | | |
|  |  |  | Solvent A: Water + 0.1% formic acid  Solvent B: Methanol+ 0.1% formic acid | | | | | | Solvent A: Water + 0.1% formic acid  Solvent B: Methanol+ ammonium acetate | | | | | |
| Step | Loop | Start  (min) | Flow  (ml/min) | Gradient | A% | B% | Description | Flow | | Gradient | A% | B% | Description |  |
| 1 | out | 0.00 | 0.75 | Step | 98 | 2 | Sample loading into the TF column | 0.25 | | Step | 50 | 50 | Analytical column conditioning |  |
| 2 | in | 2.00 | 0.13 | Step | - | 100 | Analyte transfer to analytical column | 0.12 | | Step | 100 | - | Analyte transfer to analytical column |  |
| 3 | in | 4.00 | 0.75 | Step | - | 100 | TurboFlow^TM^ column cleaning | 0.25 | | Step | 50 | 50 | LC separation |  |
| 4 | in | 5.00 | 0.75 | Step | - | 100 | TurboFlow^TM^ column and loop cleaning | 0.25 | | Ramp | 20 | 80 | LC separation |  |
| 5 | in | 7.00 | 0.75 | Step | - | 100 | TurboFlow^TM^ column and loop cleaning | 0.25 | | Step | 20 | 80 | LC separation |  |
| 6 | in | 8.00 | 0.75 | Step | - | 100 | TurboFlow^TM^ column and loop cleaning | 0.25 | | Ramp | 10 | 90 | LC separation |  |
| 7 | out | 13.00 | 0.75 | Step | - | 100 | TurboFlow^TM^ column conditioning | 0.25 | | Step | 10 | 90 | Analytical column cleaning |  |
| 8 | out | 21.00 | 0.40 | Step | - | 100 | TurboFlow^TM^ column conditioning | 0.25 | | Ramp | - | 100 | Analytical column cleaning |  |
| 9 | out | 26.00 | 0.40 | Step | - | 100 | TurboFlow^TM^ column conditioning | 0.25 | | Step | - | 100 | Analytical column cleaning |  |
| 10 | out | 38.00 | 0.40 | Step | 98 | 2 | TurboFlow^TM^ column conditioning | 0.25 | | Ramp | 50 | 50 | Analytical column conditioning |  |
| 11 | out | 39.00 | 0.75 | Step | 98 | 2 | TurboFlow^TM^ column conditioning | 0.25 | | Step | 50 | 50 | Analytical column conditioning |  |

**Table S4** Comparison of mLODs (ng/m^3^) between methods

| Sampling matrix | Cleanup | Instrumental analysis | OPEs | | PEs | | APs | | Reference |
| --- | --- | --- | --- | --- | --- | --- | --- | --- | --- |
|  |  |  | n | Range | n | Range | n | Range |  |
| QFF | - | GC-MS | 7 | 0.001-0.004 | 5 | 0.002-0.014 | 1 | 0.003 | [5] |
| QFF | Offline (Dispersive SPE) | GC-MS/MS | 12 | 0.003-0.16 | 12 | 0.003-1.13 | - | - | [6] |
| QFF | - | LC-MS/MS | - | - | 6 | 0.03-1.15 | 1 | 0.07 | [7] |
| GFF | Offline (SPE) | GC-MS | 9 | 0.003-0.12 | 8 | 0.007-0.23 | - | - | [8] |
| GFF | - | GC-MS | - | - | 14 | 0.007-0.13 | - | - | [9] |
| QFF | - | GC-MS | - | - | 7 | 0.02-13.0 | 8 | 0.01-4.60 | [10] |
| SPE cartridge | Online (TFC) | LC-MS/MS | 15 | 0.02-0.33 | 11 | 0.02-1.94 | 7 | 0.07-1.22 | This study |


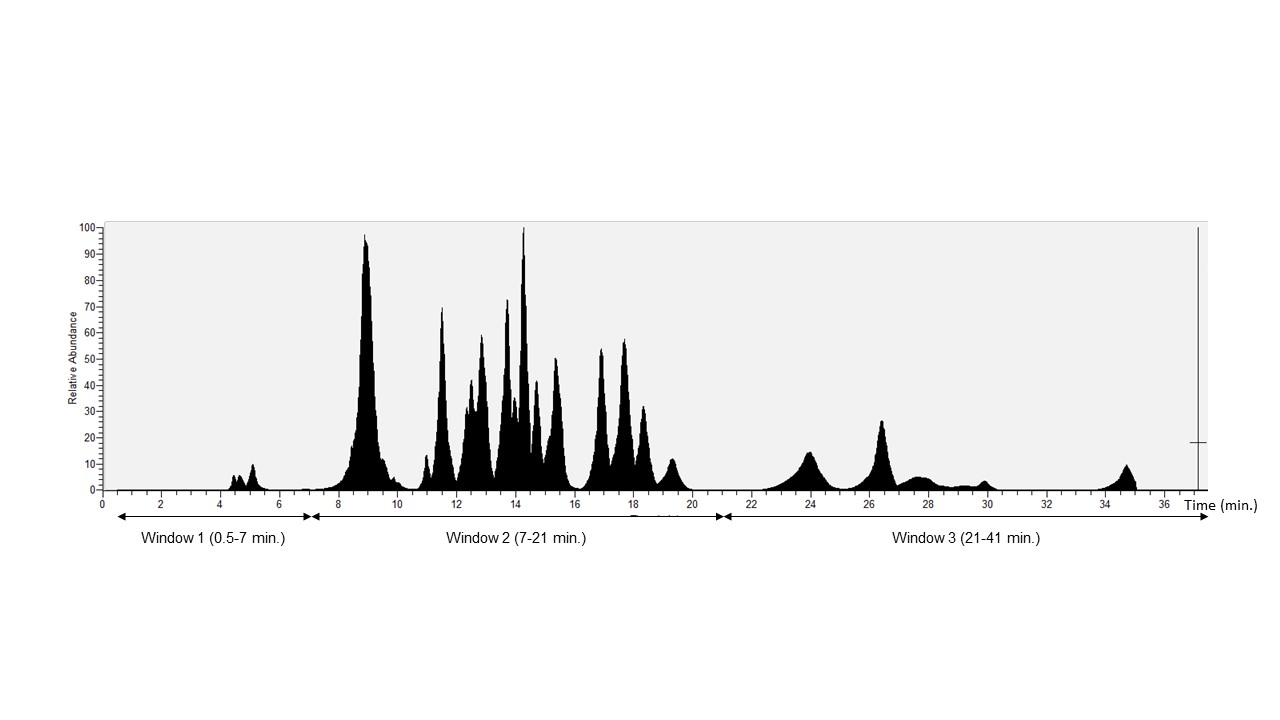


Window 1 (0.5-7 min.)


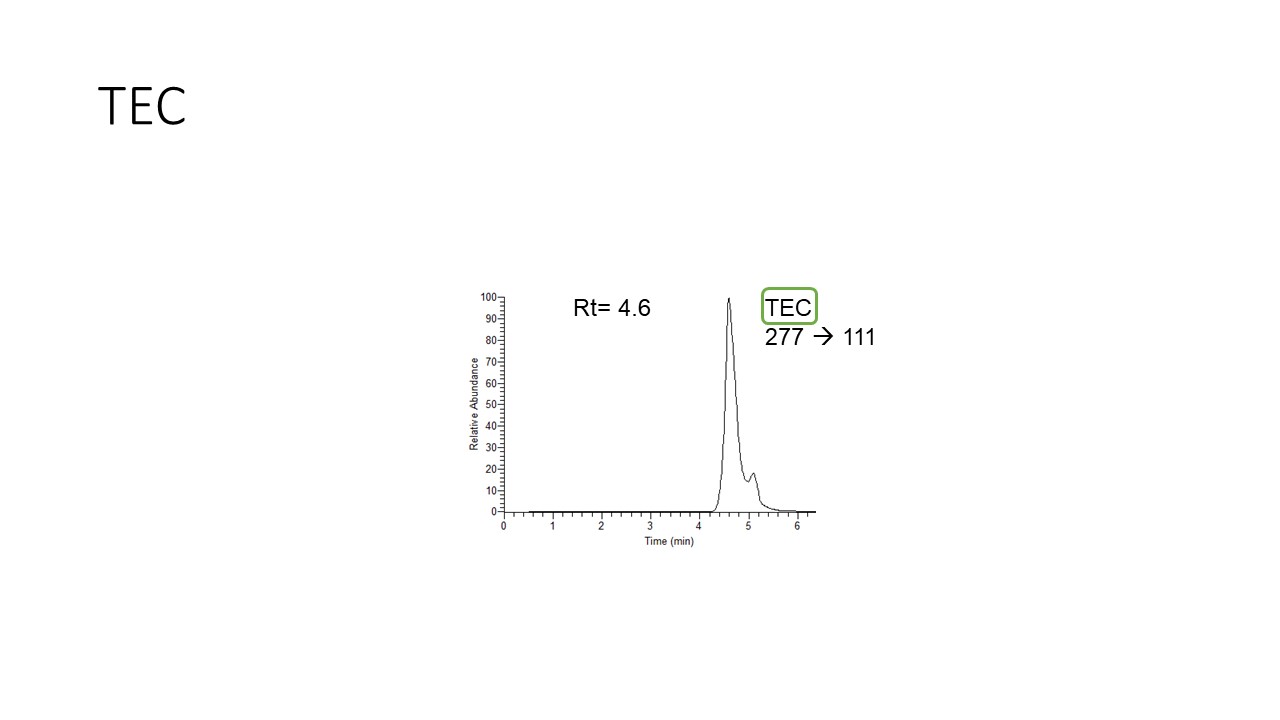

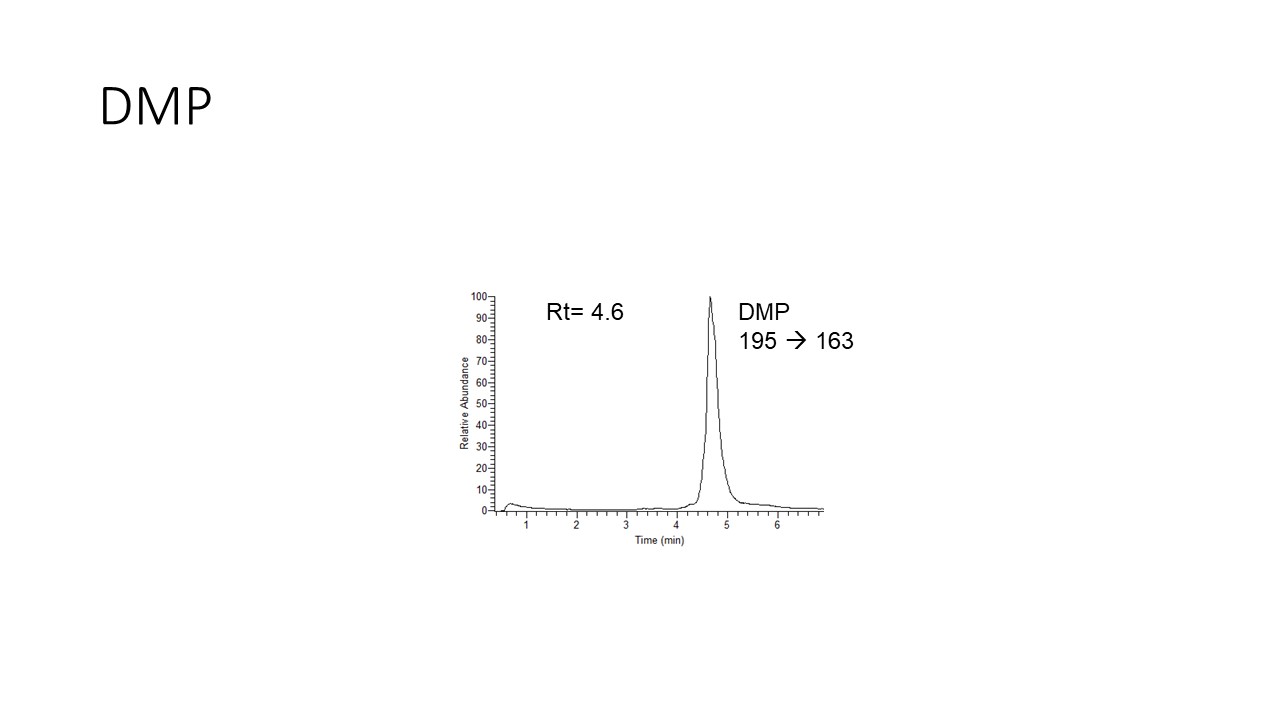

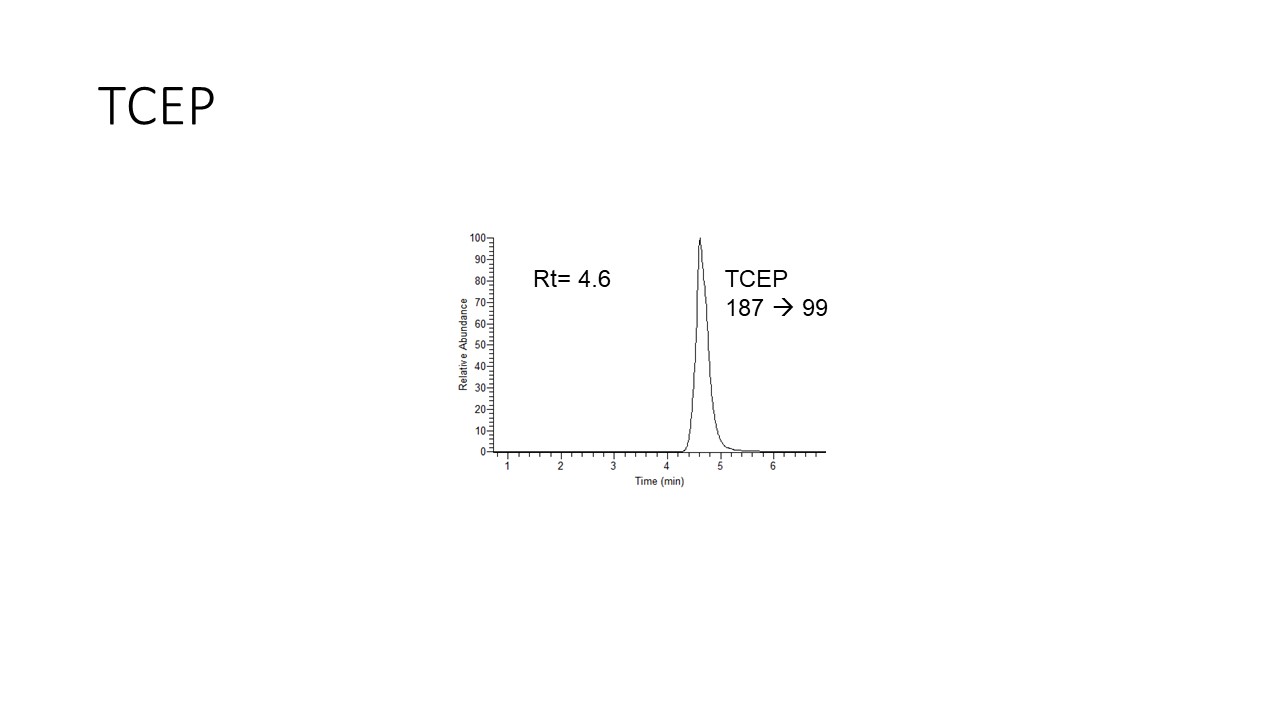

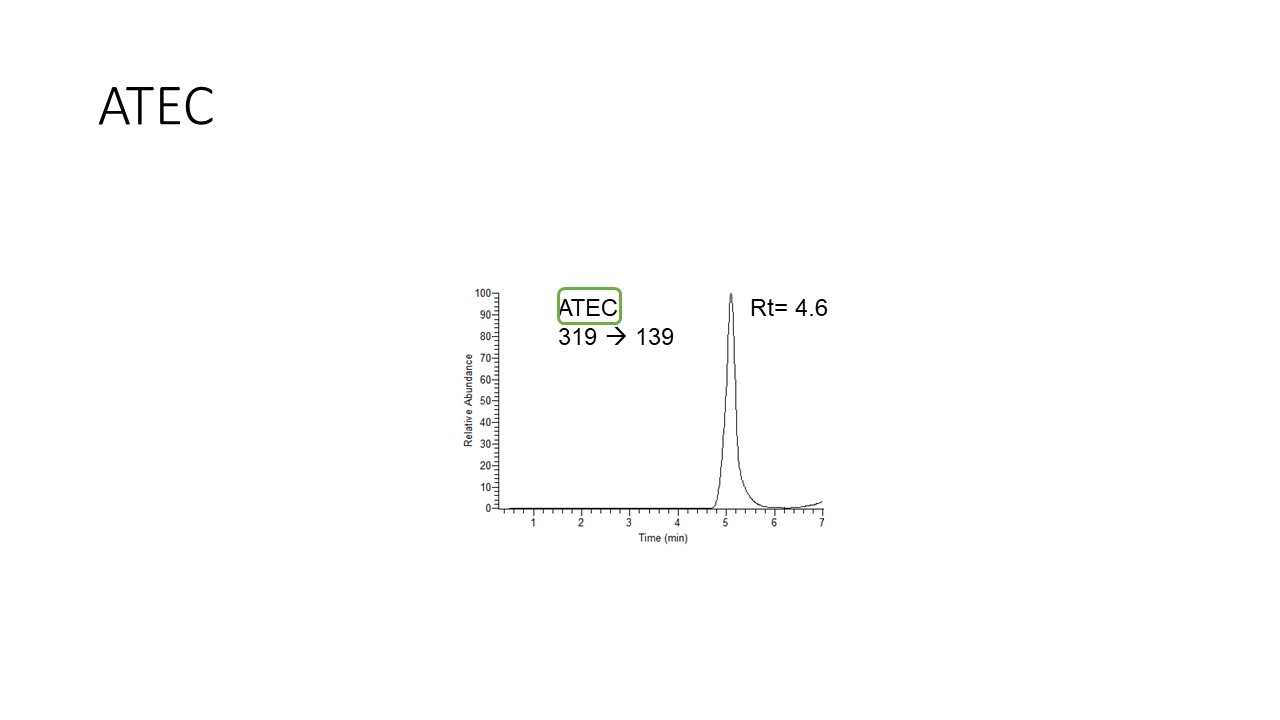


Window 2 (7-21 min.)


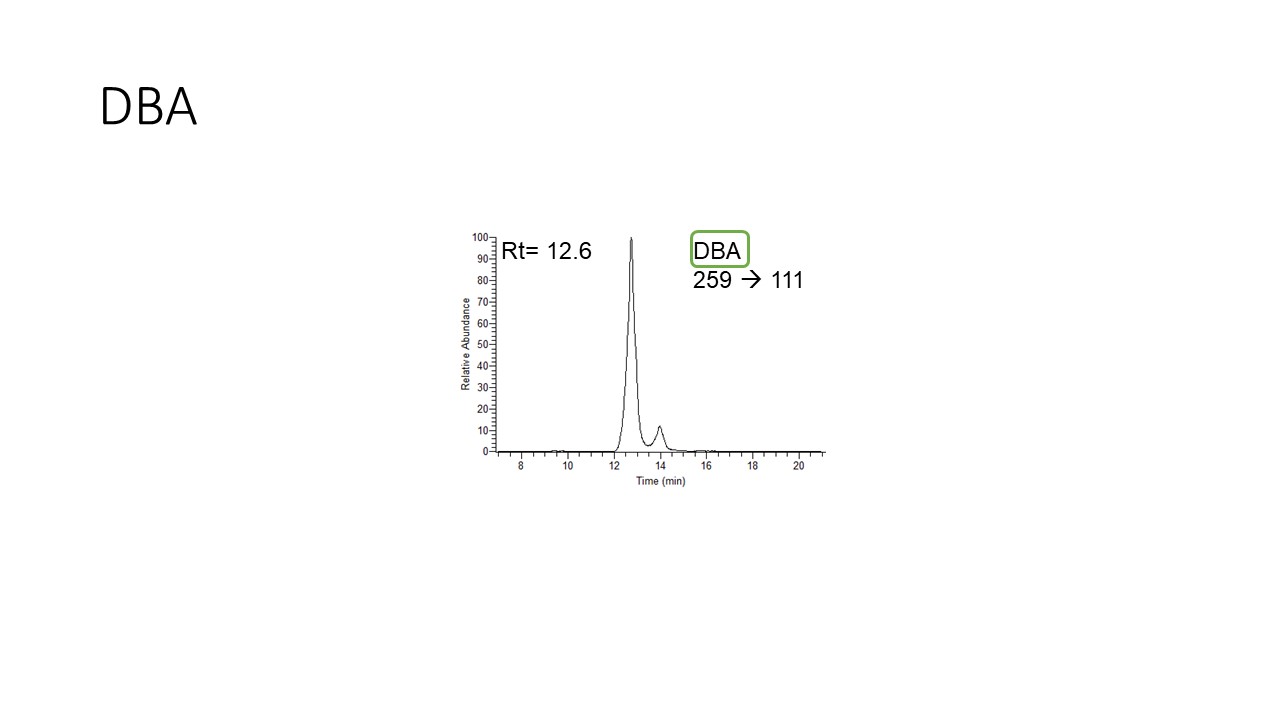

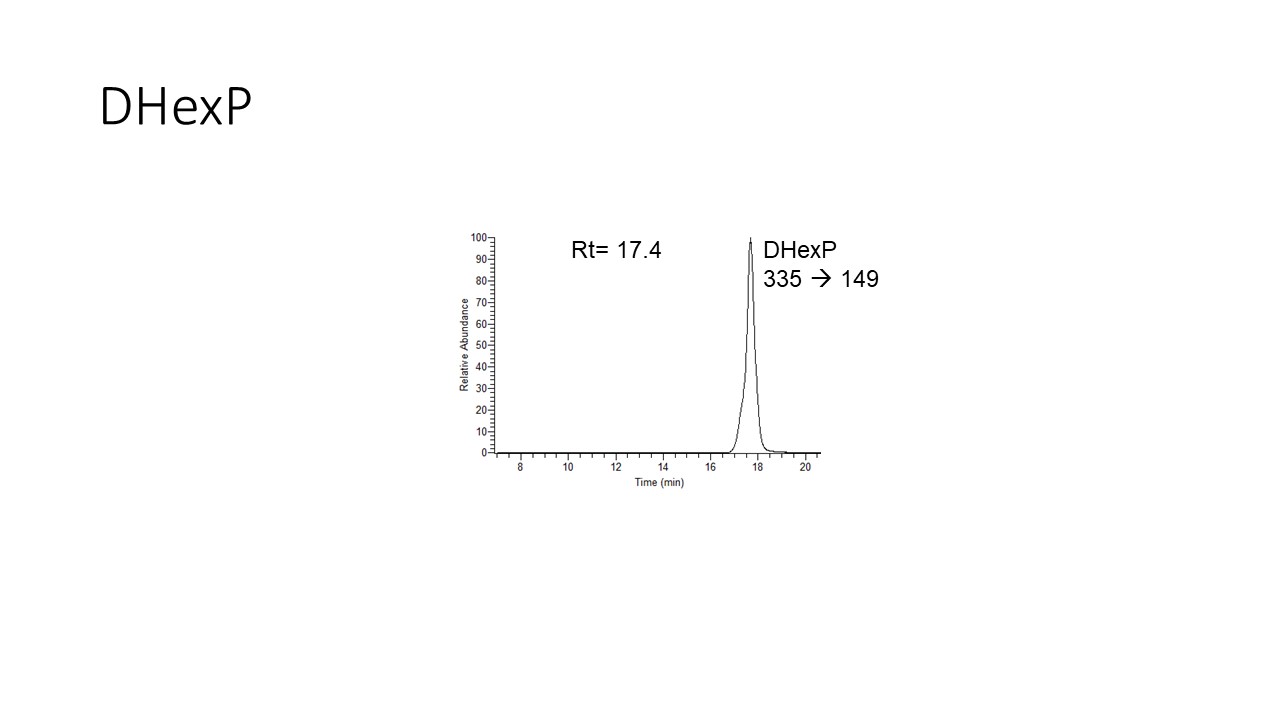

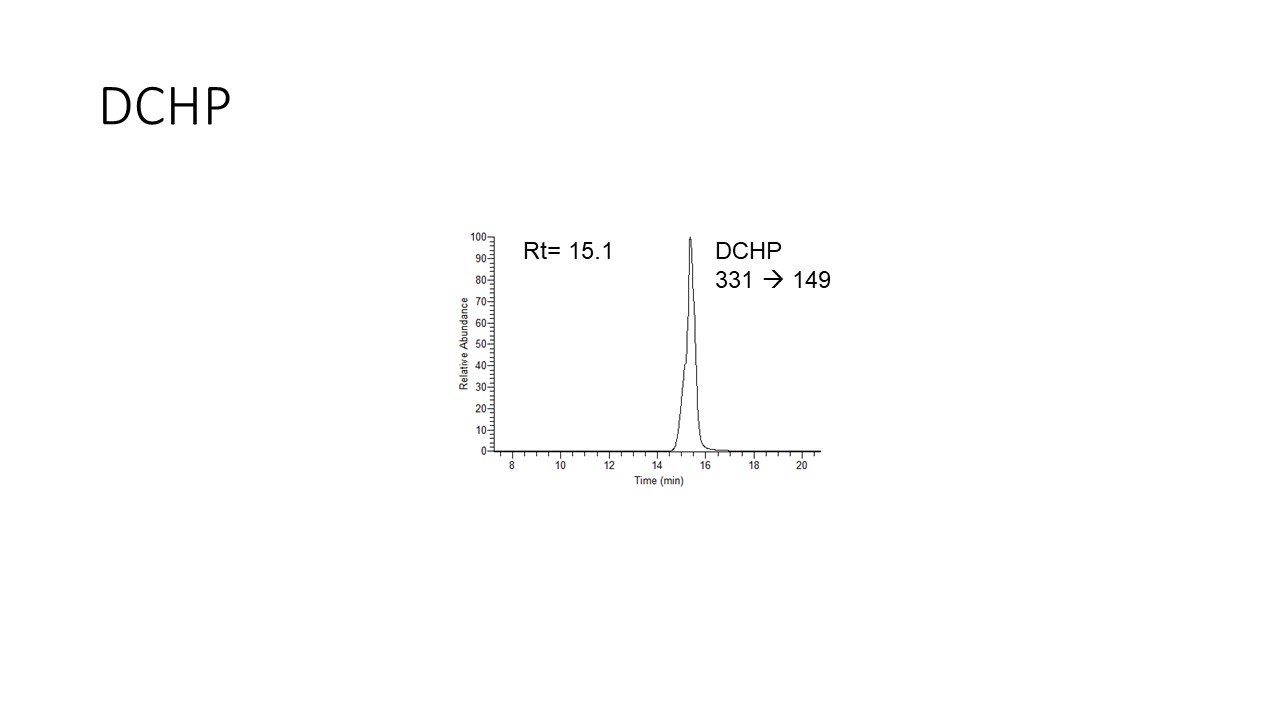

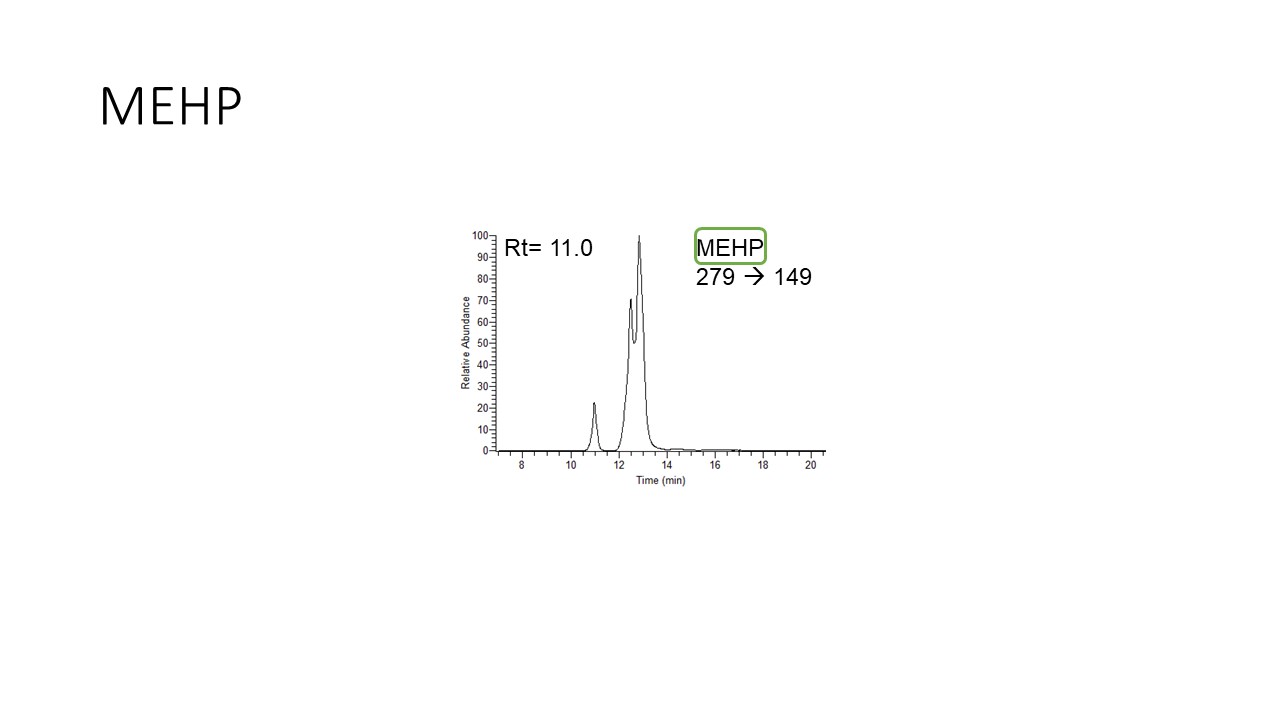

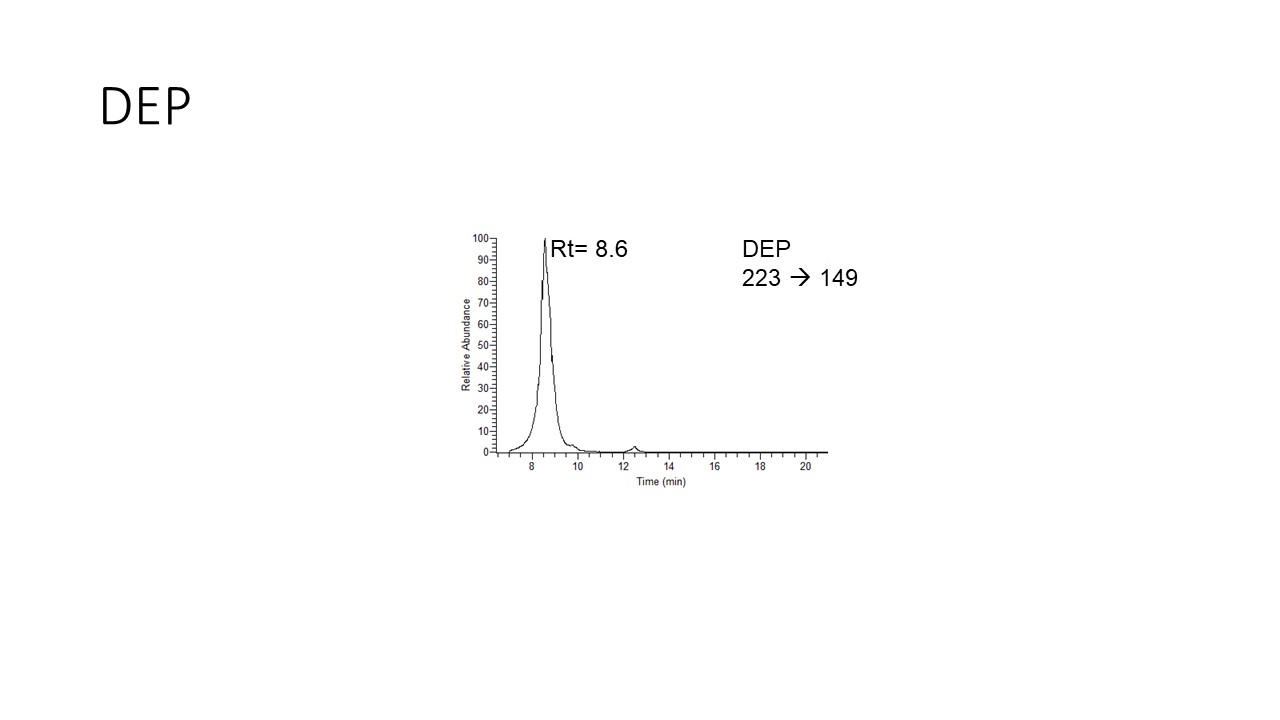

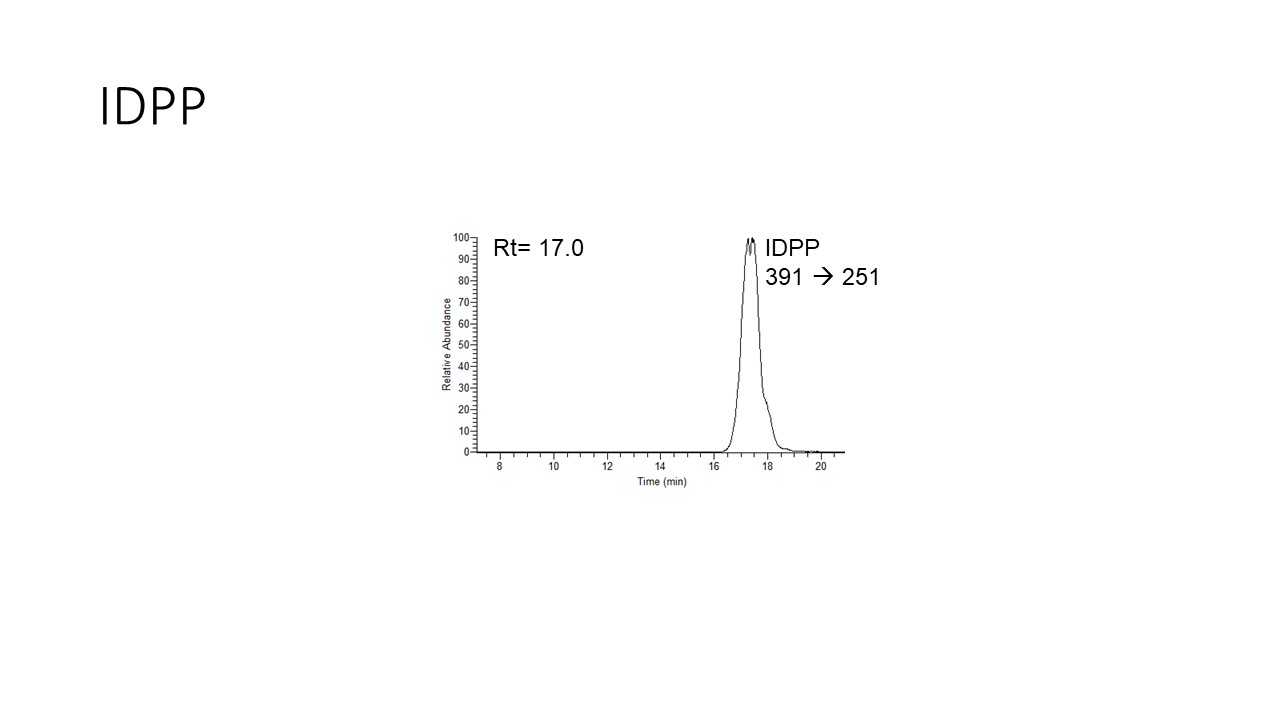

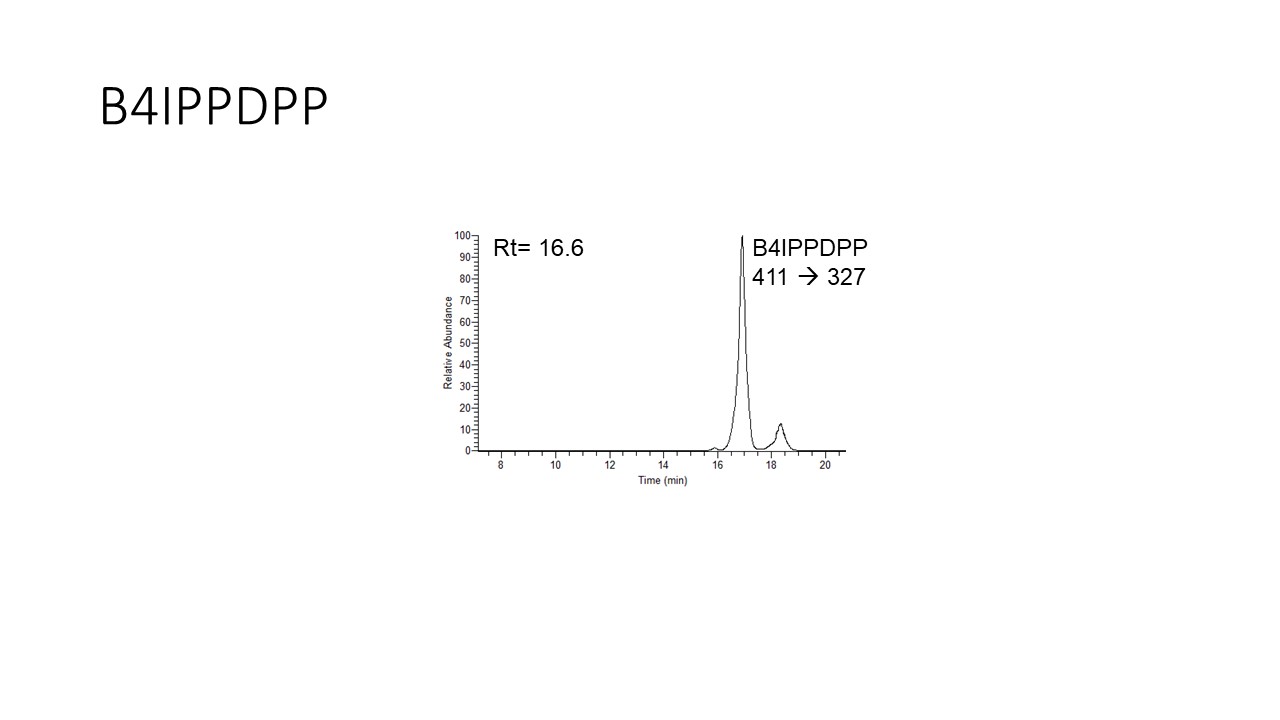

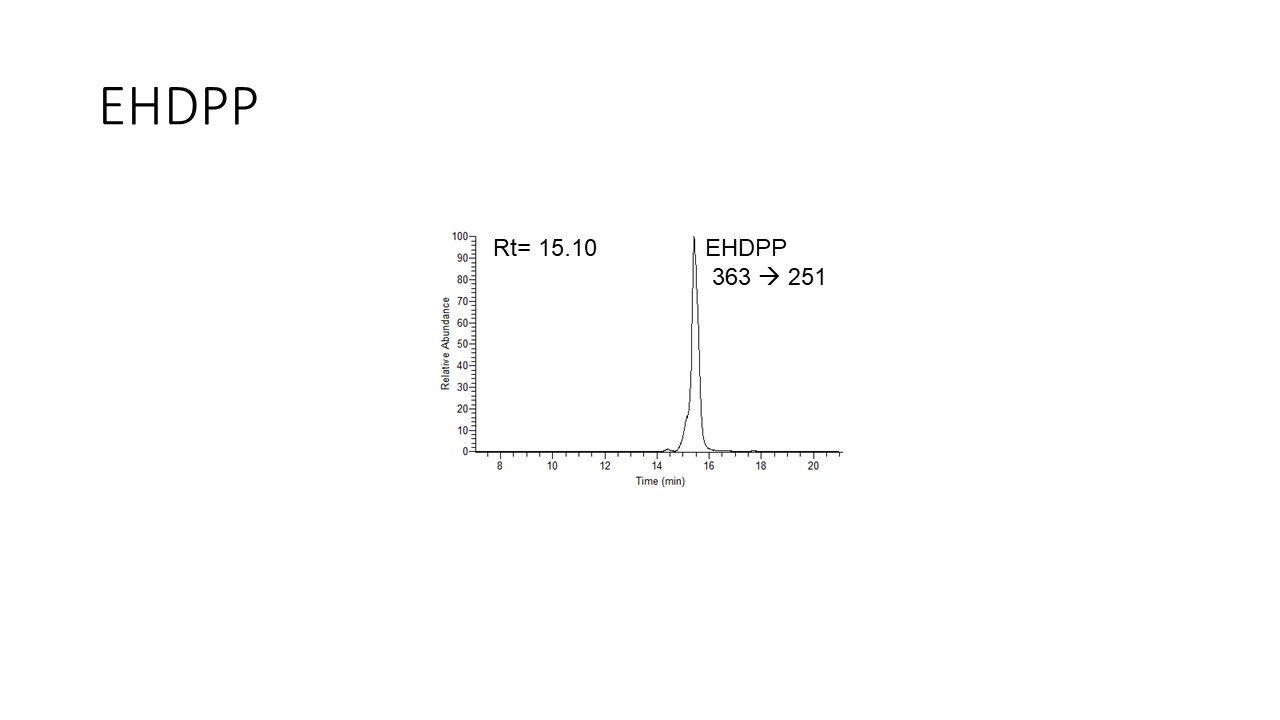

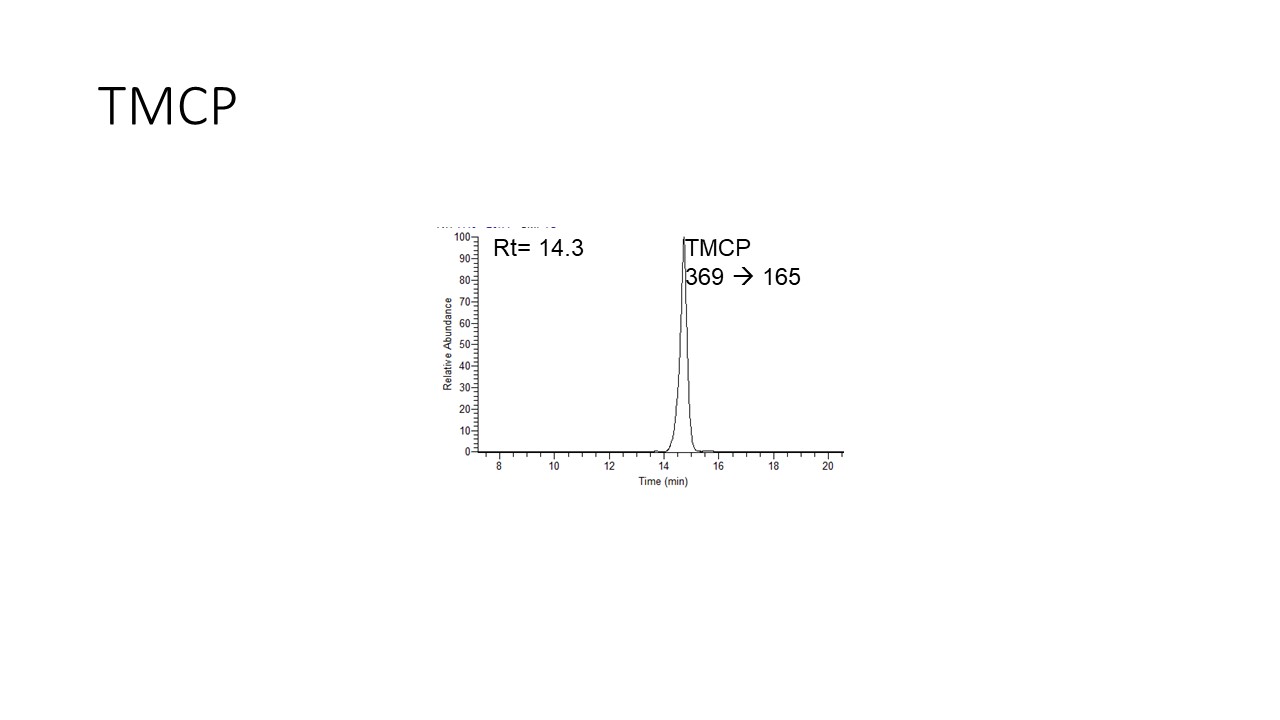

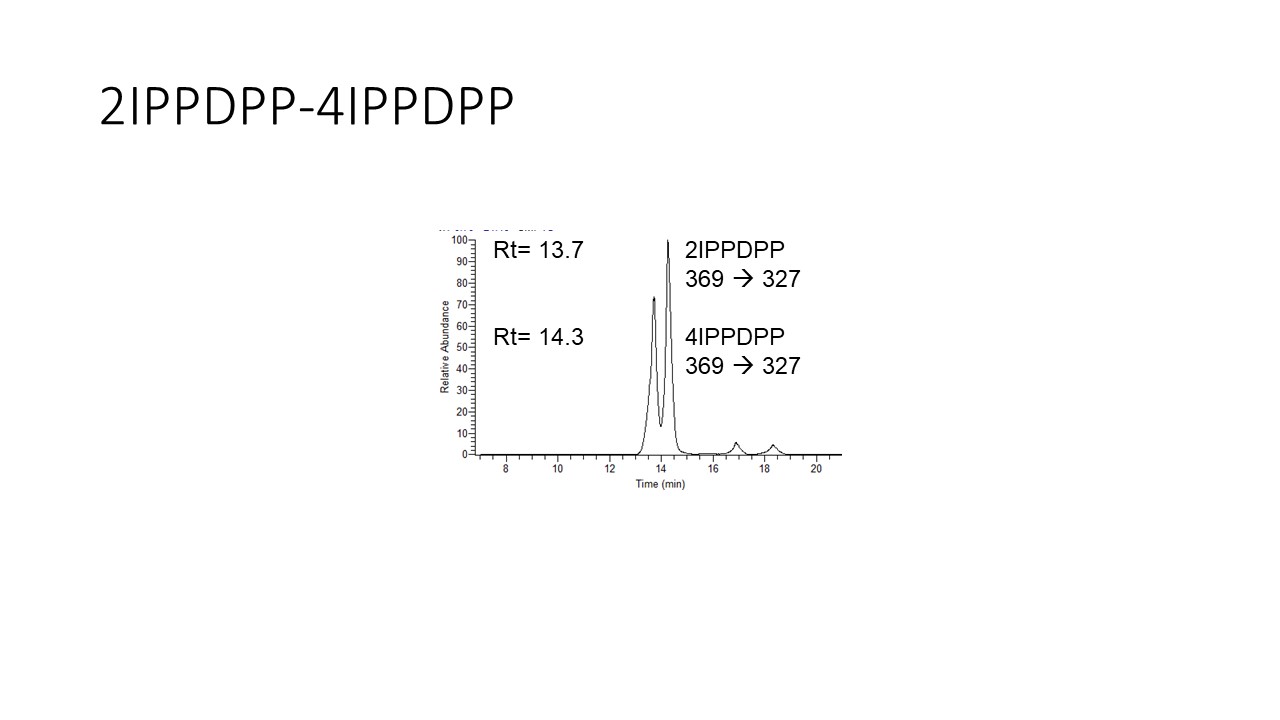

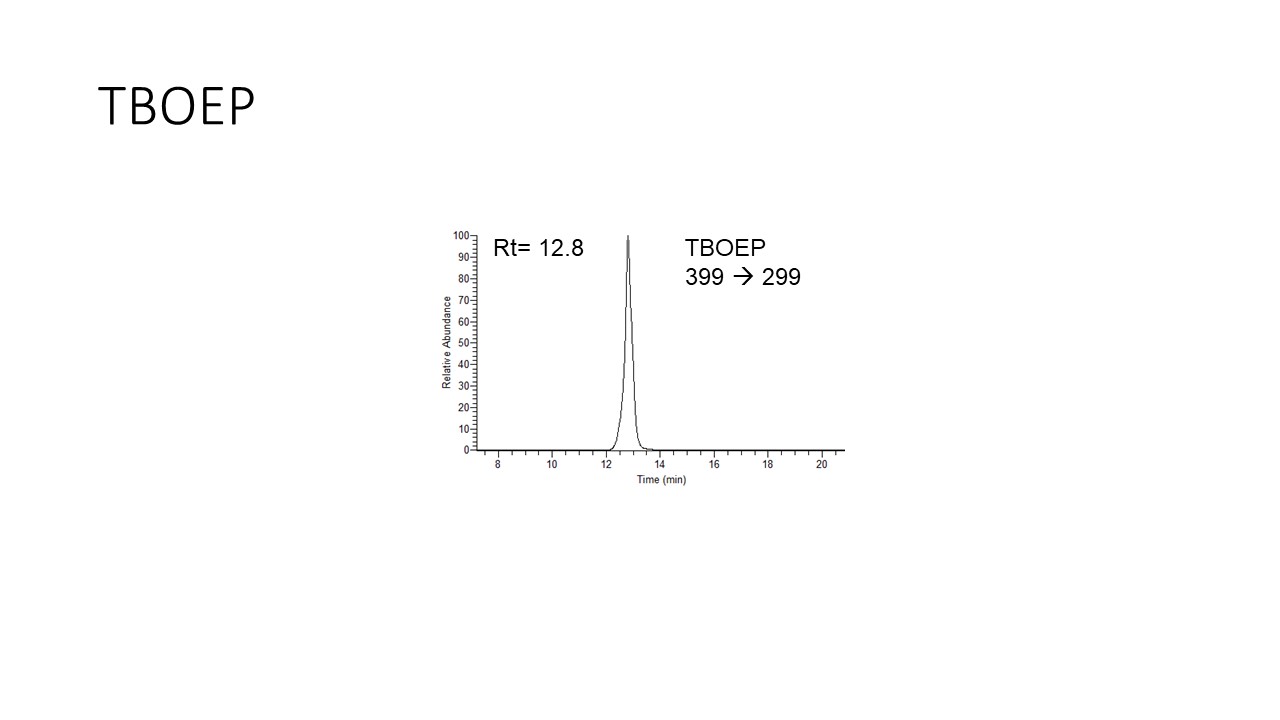

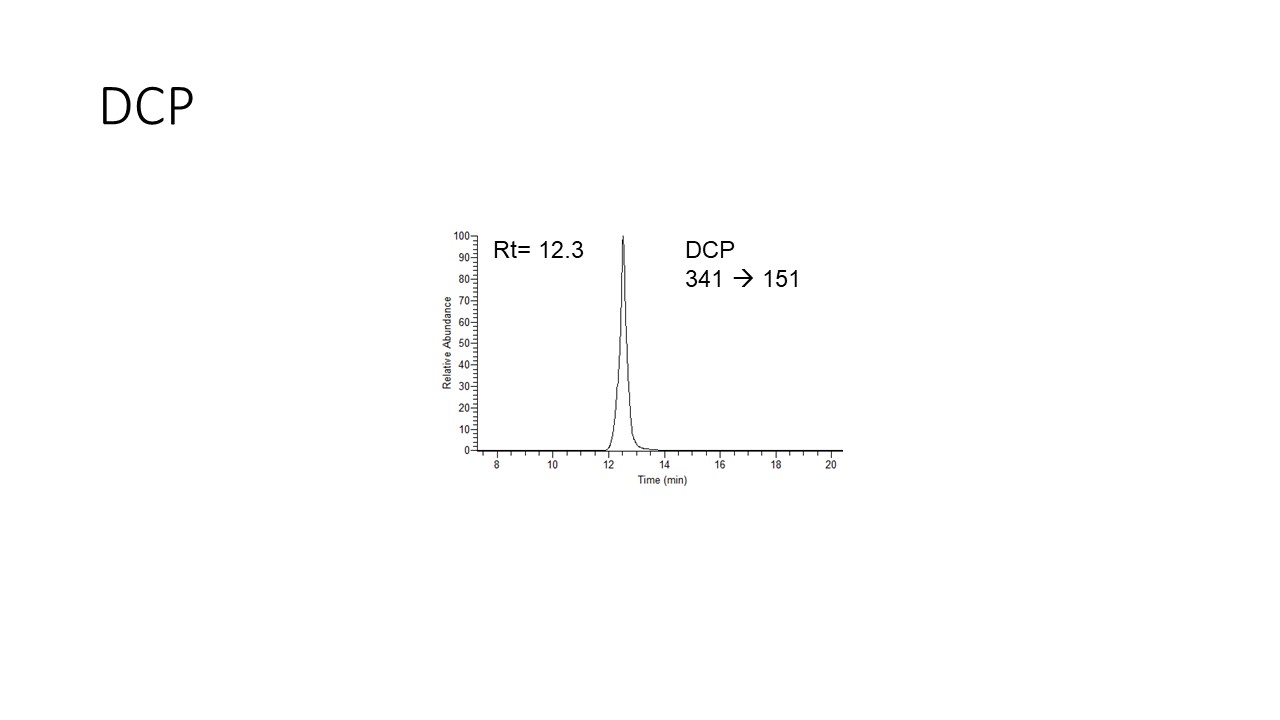

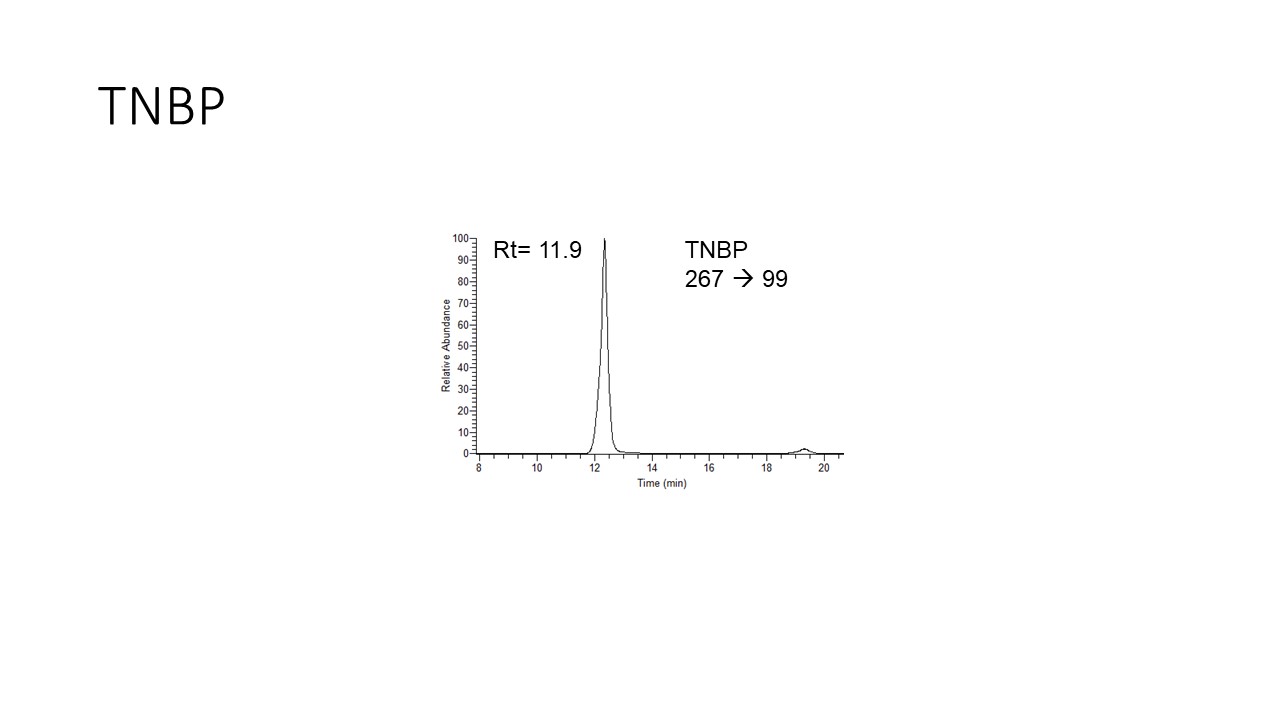

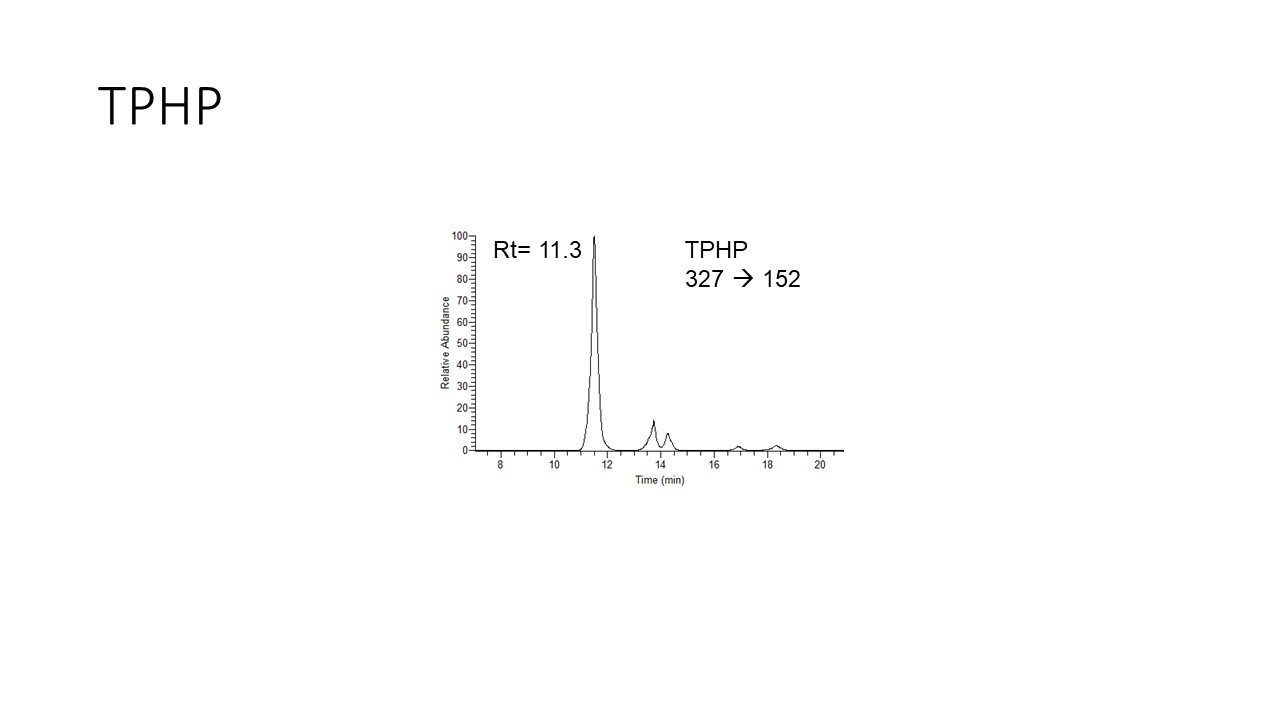

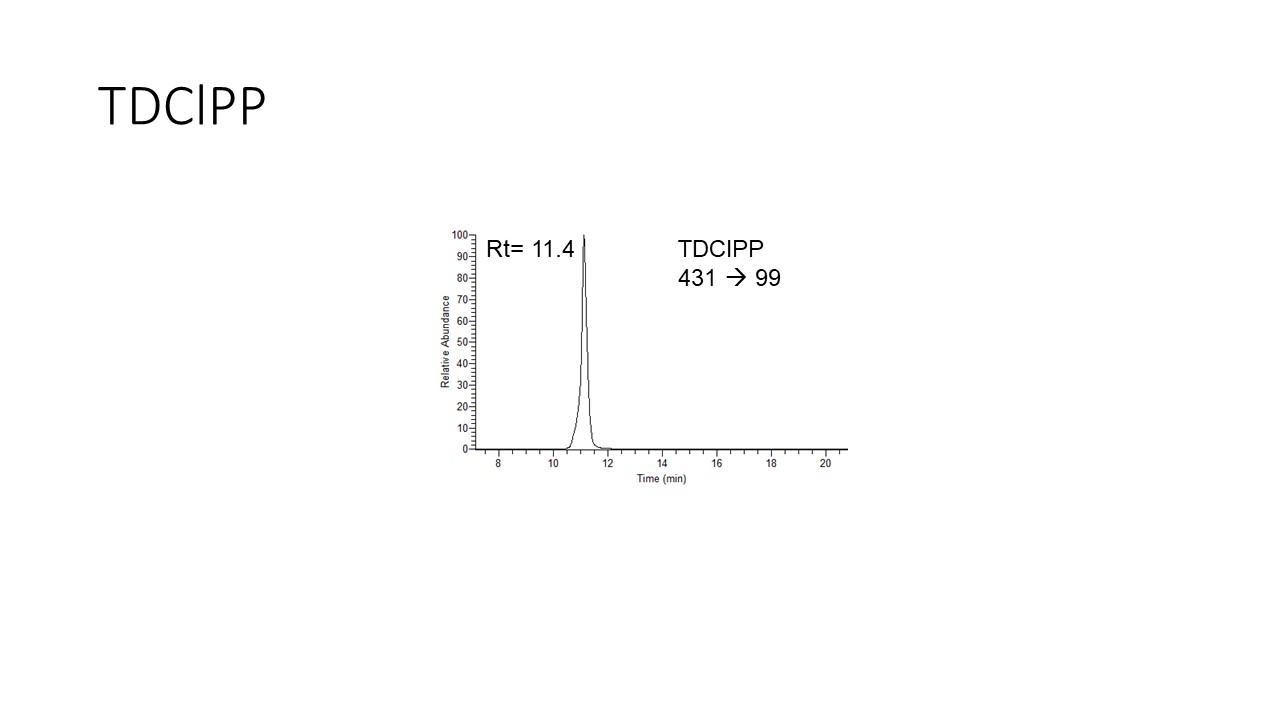

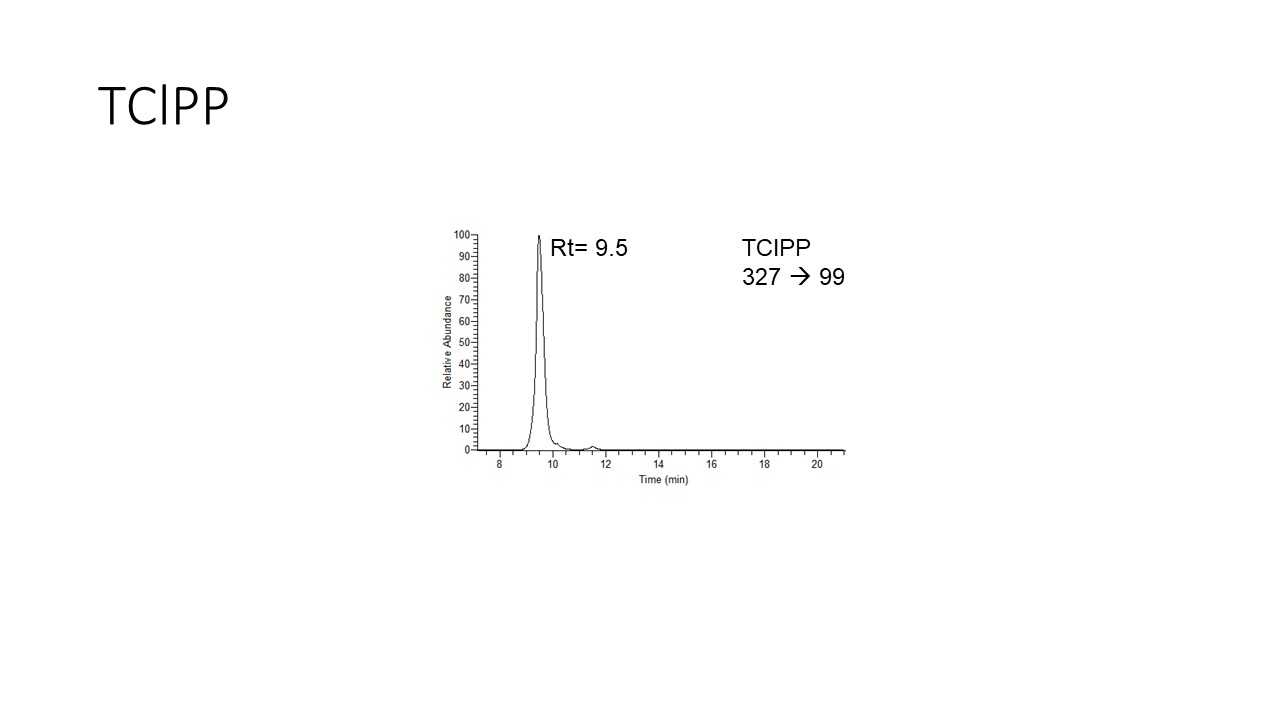

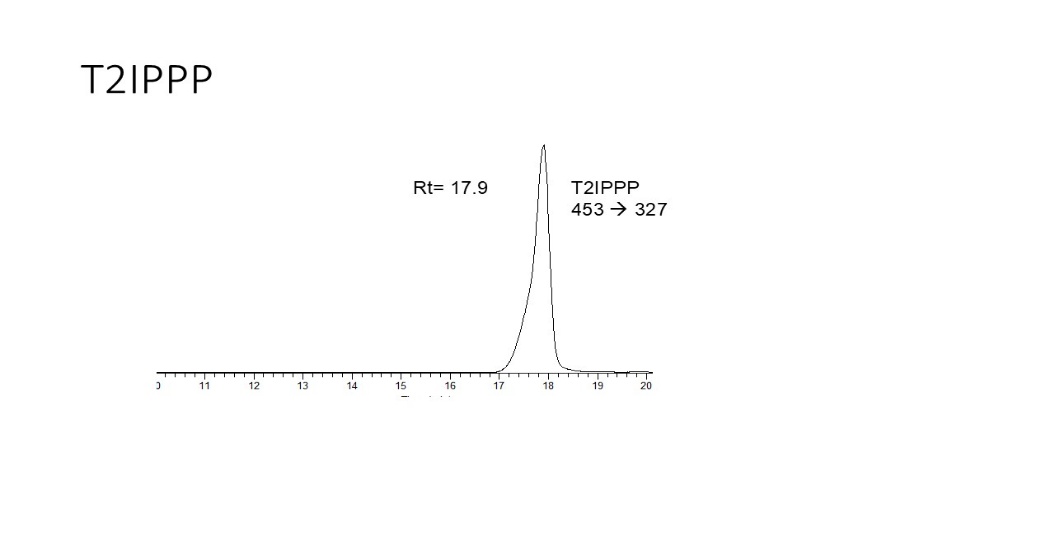

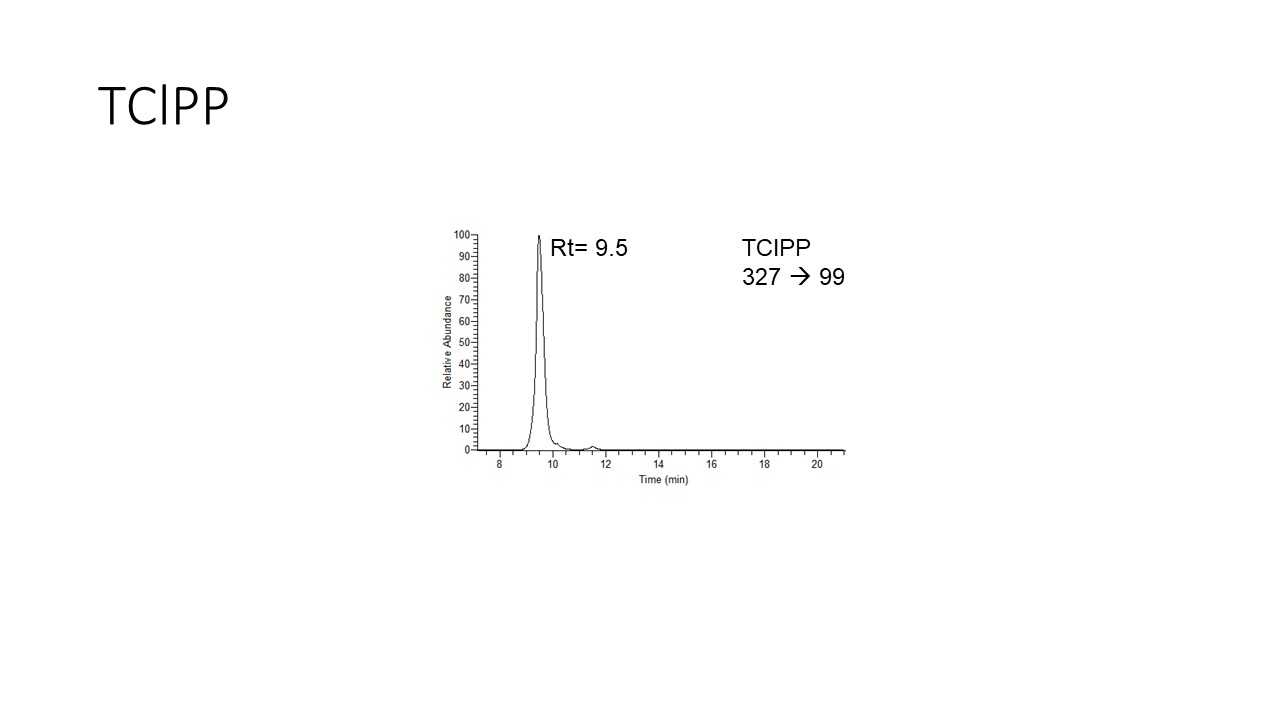

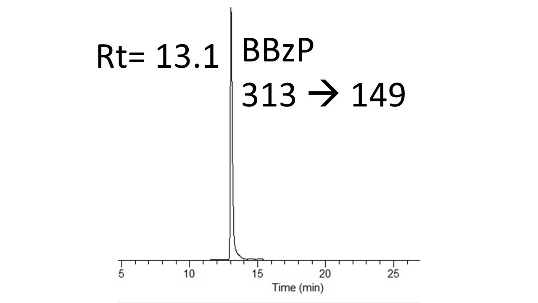


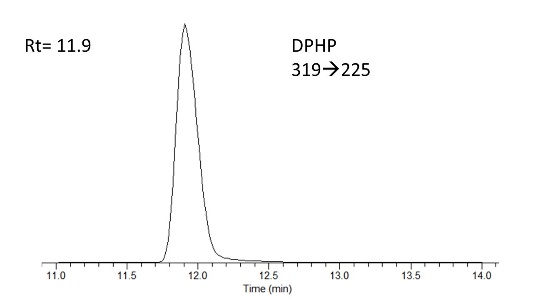


Window 3 (21-38 min.)


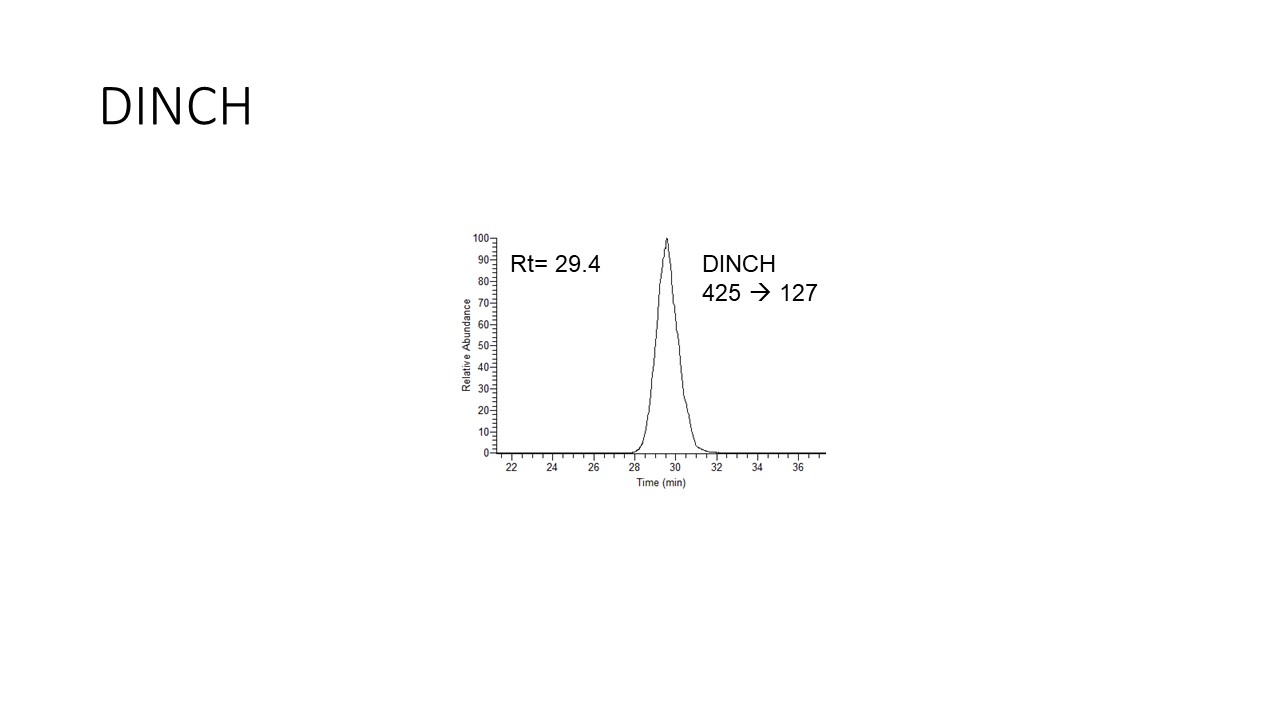

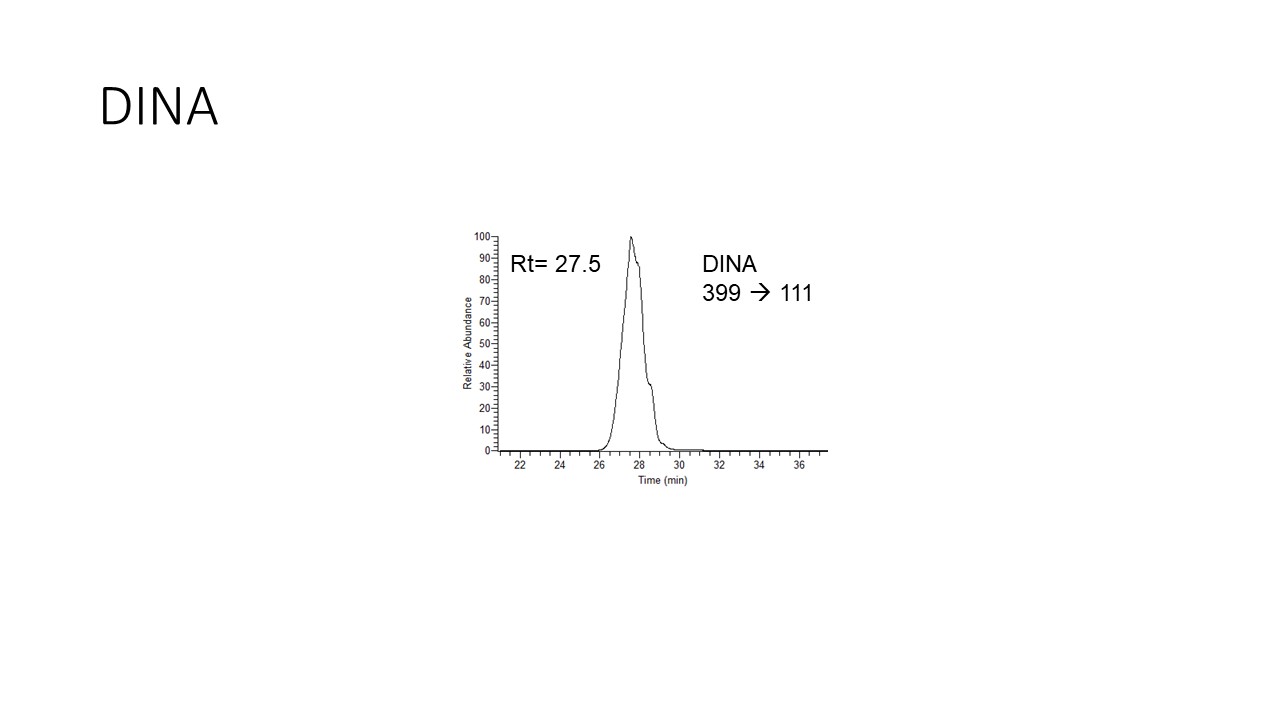

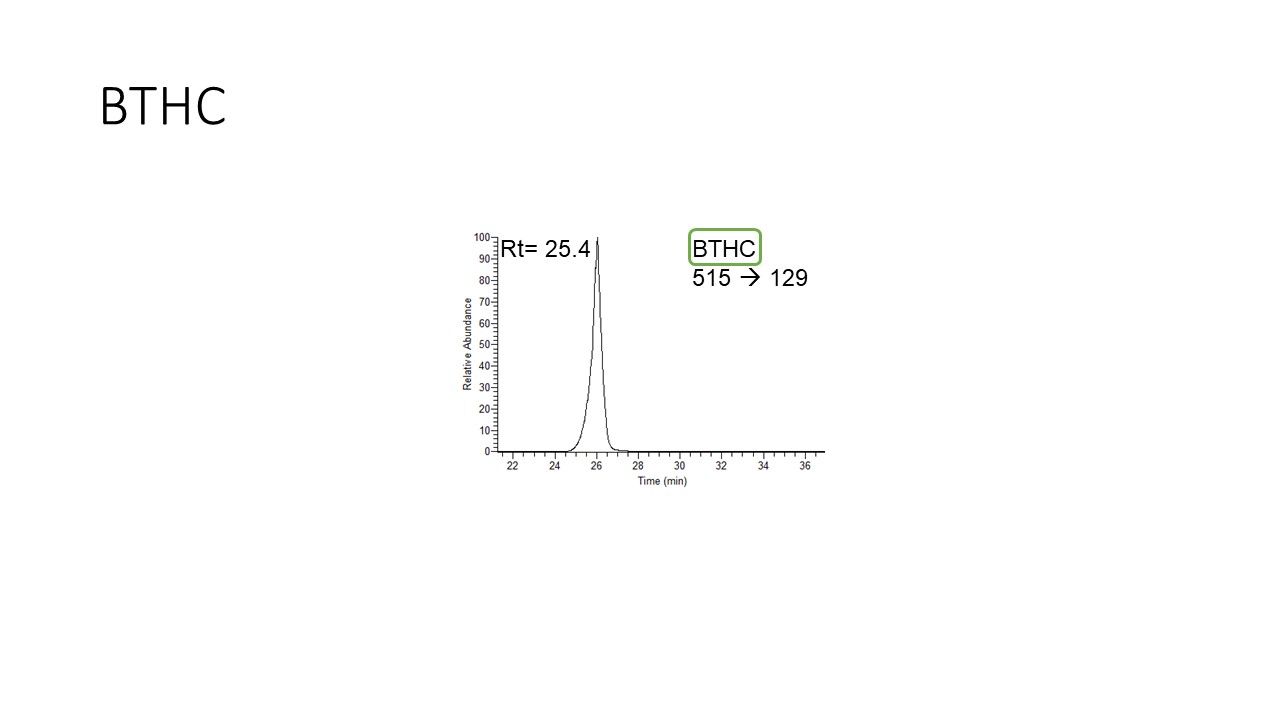

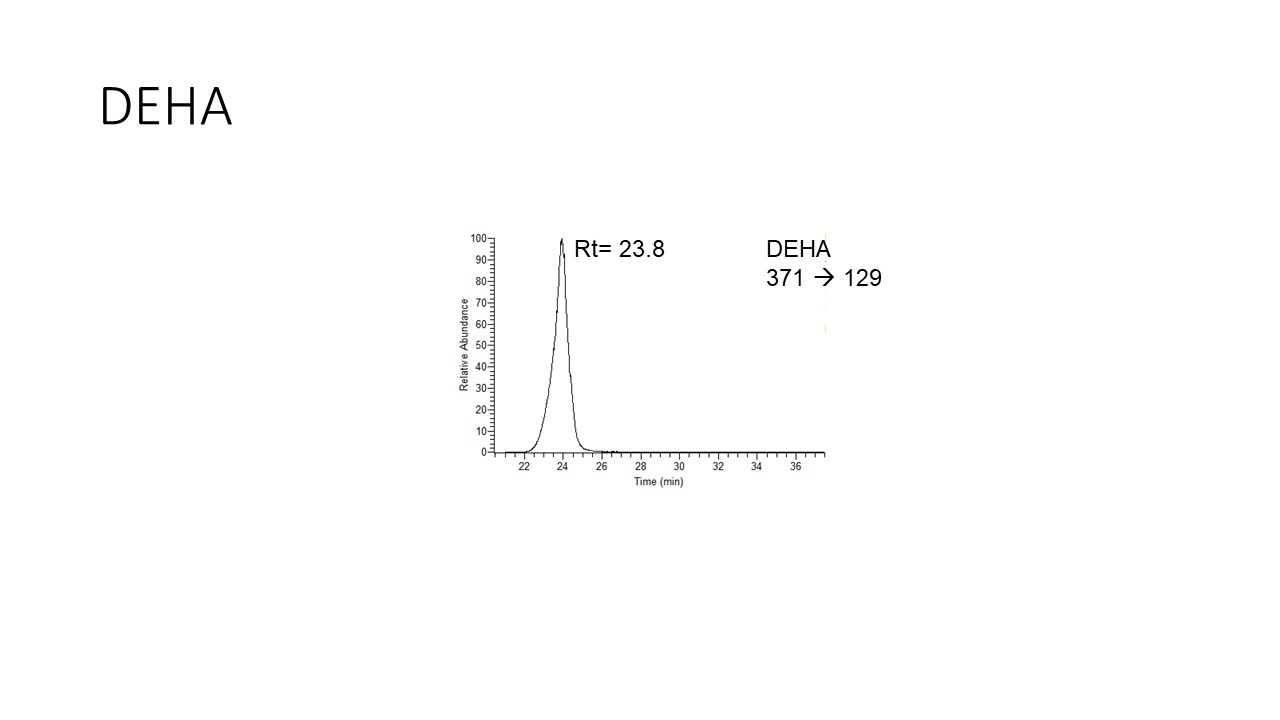

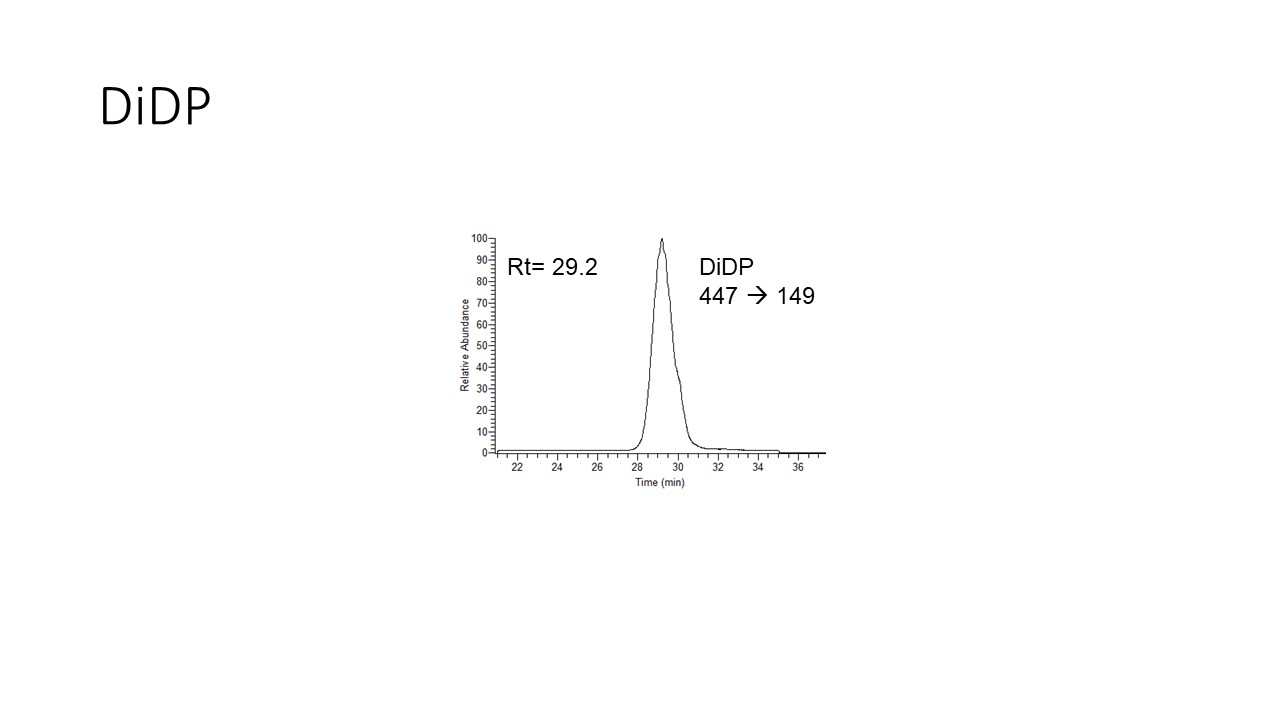

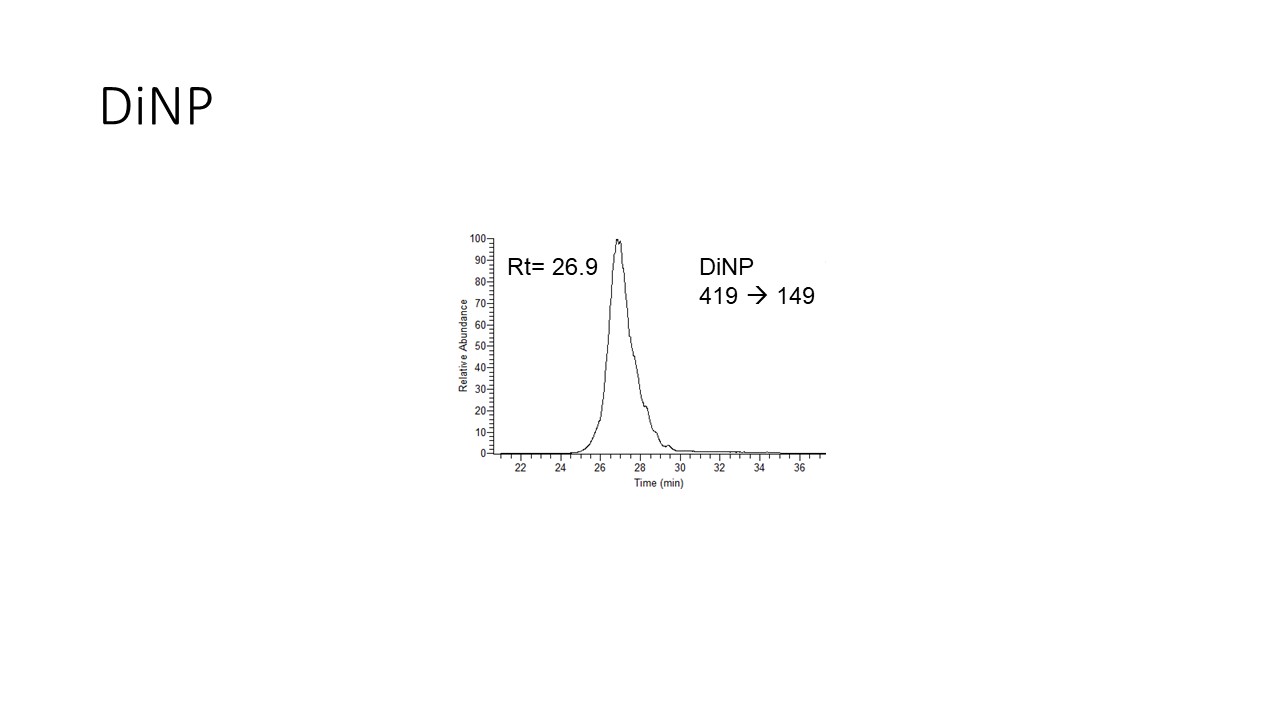


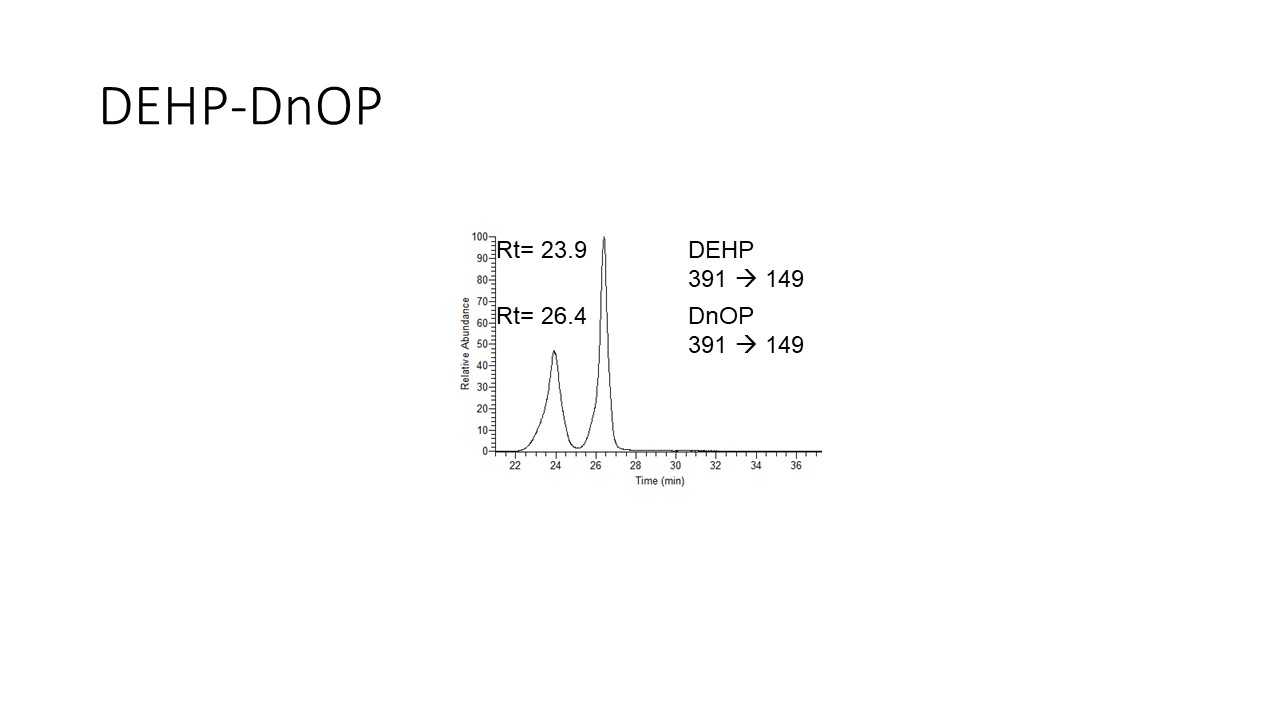

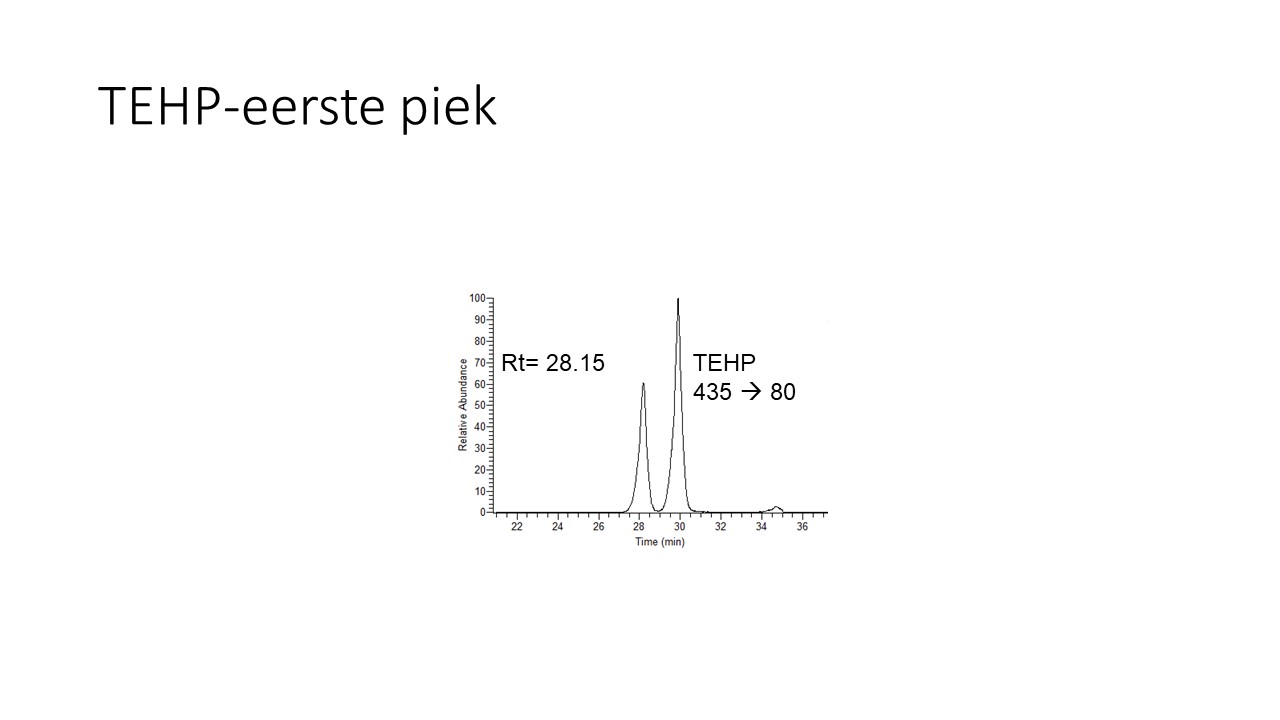

**Fig. S2** LC-MS/MS chromatograms of 33 quantifiable FRs (SRM1 transition shown per window) and plasticizers in a high recovery sample; in green the added analytes.

**Fig. S4** Percentage of detected plasticizers (OPEs and APs) included in the method compared to initial conditions (T0). Top-bottom: storage at room temperature, 4ºC and -20ºC. T0 (black): extracted on the day of the spike (control); Tw1, Tw2, Tm3 extracted respectively, one week, two weeks, and three months after spiking.

**Fig. S5** Percentage of detected plasticizers (PEs) included in the method compared to initial conditions (T0). Top-bottom: storage at room temperature, 4ºC and -20ºC. T0 (black): extracted on the day of the spike (control); Tw1, Tw2, Tm3 extracted respectively, one week, two weeks, and three months after spiking.

**Table S5** OPEs, PEs and APs concentrations (ng/m^3^) of indoor air samples of different sampling locations.

|  | Locations | | | |
| --- | --- | --- | --- | --- |
|  | Warehouse (ng/m^3^) | Office 1 (ng/m^3^) | Office 2 (ng/m^3^) | Office 3 (ng/m^3^) |
| OPEs | | | | |
| TEP | 10.5 | 8.50 | 1.60 | 23.5 |
| TClPP | 4.10 | 3.7 | 3.14 | 3.80 |
| TPHP | nq | 0.04 | nq | nq |
| TNBP | 0.74 | 0.37 | 0.04 | 0.39 |
| DCP | 0.05 | 0.06 | nd | nd |
| 2IPPDPP | 0.38 | 0.82 | 0.34 | 0.57 |
| 4IPPDPP | nd | nd | nd | nd |
| TmCP | 0.04 | 0.03 | 0.04 | 0.02 |
| EHDPP | nq | nq | nq | nq |
| ∑OPEs | 15.8 | 13.5 | 5.15 | 28.2 |
| PEs | | | | |
| DMP | 5.64 | 3.04 | 6.44 | 9.91 |
| DEP | nq | 12.0 | nd | 15.7 |
| DiBP+DnBP | 20.8 | 41.9 | 14.4 | 35.0 |
| BBzP | 0.22 | 0.30 | 0.10 | 0.83 |
| DCHP | 0.02 | 0.01 | 0.01 | 0.01 |
| DEHP | nd | 2.21 | nd | 8.43 |
| ∑PEs | 26.7 | 59.5 | 21.0 | 69.9 |
| APs | | | | |
| TEC | 23.8 | 27.2 | 4.94 | 28.9 |
| DIPA | nd | 0.37 | nd | nd |
| TBC | nq | nq | nd | 0.26 |
| DBA | 7.33 | 7.40 | 5.59 | 1.87 |
| ATBC | nq | 0.07 | nd | 0.12 |
| DEHA | 4.04 | nq | nq | nq |
| ∑APs | 35.2 | 35.1 | 10.5 | 31.1 |
| **∑Plasticizers** | **77.7** | **108** | **36.6** | **129** |

nd: peak not detected (concentrations below mLOD); nq: not quantified (concentrations below mLOQ).

**References**

1. USEPA. Regional Screening Level (RSL) Summary Table [Internet]. 2019 [cited 2023 Jun 21]. p. 11. Available from: https://semspub.epa.gov/work/HQ/404057.pdf

2. Li J, Zhang Z, Ma L, Zhang Y, Niu Z. Implementation of USEPA RfD and SFO for improved risk assessment of organophosphate esters (organophosphate flame retardants and plasticizers). Environ Int. 2018 May 1;114:21–6.

3. He C, Wang X, Thai P, Baduel C, Gallen C, Banks A, et al. Organophosphate and brominated flame retardants in Australian indoor environments: Levels, sources, and preliminary assessment of human exposure. Environ Pollut. 2018 Apr 1;235:670–9.

4. Fernández-Arribas J, Callejas-Martos S, Balasch A, Moreno T, Eljarrat E. Simultaneous analysis of several plasticizer classes in different matrices by on-line turbulent flow chromatography-LC-MS/MS. Anal Bioanal Chem. 2024 Dec;416(29):6957–72.

5. Maceira A, Pecikoza I, Marcé RM, Borrull F. Multi-residue analysis of several high-production-volume chemicals present in the particulate matter from outdoor air. A preliminary human exposure estimation. Chemosphere. 2020;252.

6. Sánchez-Piñero J, Moreda-Piñeiro J, Moscoso-Pérez C, FernándezGonzález V, Prada-Rodríguez D, López-Mahía P. Development and validation of a multi-pollutant method for the analysis of polycyclic aromatic hydrocarbons, synthetic musk compounds and plasticizers in atmospheric particulate matter (PM2.5). Talanta Open. 2021;4(May).

7. Patnana DP, Chandra BP, Chaudhary P, Sinha B, Sinha V. Optimized LC-MS/MS method for simultaneous determination of endocrine disruptors and PAHs bound to PM2.5: Sources and health risk in Indo-Gangetic Plain. Atmos Environ [Internet]. 2022;290(June):119363. Available from: https://doi.org/10.1016/j.atmosenv.2022.119363

8. Wang Y, Zhang Z, Xu Y, Rodgers TFM, Ablimit M, Li J, et al. Identifying the contributions of root and foliage gaseous/particle uptakes to indoor plants for phthalates, OPFRs and PAHs. Sci Total Environ [Internet]. 2023;883(April):163644. Available from: https://doi.org/10.1016/j.scitotenv.2023.163644

9. Lu H, Chen D, Zhu Z, Yang L, Huang L, Xu C, et al. Atmospheric phthalate esters in a multi-function area of Hangzhou: Temporal variation, gas/particle phase distribution, and population exposure risk. Sci Total Environ [Internet]. 2023;894(April):163987. Available from: https://doi.org/10.1016/j.scitotenv.2023.163987

10. Huo CY, Li WL, Liu LY, Sun Y, Guo JQ, Wang L, et al. Seasonal variations of airborne phthalates and novel non-phthalate plasticizers in a test residence in cold regions: Effects of temperature, humidity, total suspended particulate matter, and sources. Sci Total Environ. 2023 Mar 10;863:160852.
